# Mapping the biological effects of micro- and nanoplastics in mammalian intestinal models: a scoping review

**DOI:** 10.1186/s12876-026-05475-0

**Published:** 2026-10-27

**Authors:** Deniz Özkan Vardar, Necati Vardar

**Affiliations:** 1 Department of Pharmacy Services, Vocational School of Health Services, Lokman Hekim University, 06510 Ankara, Türkiye, Turkey; 2 Department of Electrical and Electronics Engineering, Faculty of Engineering and Natural Sciences, KTO Karatay University, 42020 Konya, Türkiye, Turkey

**Keywords:** Nanotechnology, Micro-nano plastics, Intestinal toxicity, Gastrointestinal tract, Mammalian models, Scoping review

## Abstract

**Background:**

Micro- and nanoplastics (MNPLs) are increasingly recognized as emerging environmental contaminants with potential implications for gastrointestinal health. Although experimental research on intestinal MNPL toxicity has expanded rapidly, the available evidence remains highly heterogeneous with respect to experimental models, polymer composition, particle size, particle condition, exposure scenarios, and biological endpoints. Therefore, a structured evidence map is needed to clarify dominant methodological patterns and identify key knowledge gaps in the field.

**Methods:**

This study was conducted as a scoping review in accordance with the Joanna Briggs Institute framework and the PRISMA-ScR guidelines. A comprehensive search was performed in PubMed, Scopus, and Web of Science to identify eligible original in vitro, ex vivo, and in vivo experimental research investigating the biological effects of MNPLs in mammalian intestinal models. Data were extracted and synthesized based on experimental models, particle characteristics, exposure scenarios, and toxicological outcomes.

**Results:**

A total of 90 peer-reviewed studies were included from 3,540 initially identified records. The evidence base was strongly dominated by simplified experimental systems, with in vitro studies accounting for 88.9% of the included studies (*n* = 80/90). Caco-2-based systems represented the dominant experimental platform overall (52.2%, *n* = 47/90), while Caco-2 monoculture-like models accounted for 36.7% (*n* = 33/90). A pronounced polymer bias was observed, with polystyrene (PS) appearing in 60.0% of the included studies (*n* = 54/90). Nanoplastics were the most frequently investigated size category (60.0%, *n* = 54/90), and most studies relied on virgin particles (68.9%, *n* = 62/90). Across the literature, the most prominent evidence themes included oxidative stress and mitochondrial injury, uptake and translocation, barrier dysfunction and permeability changes, inflammation and immune signaling, and cell death- or DNA damage-related responses.

**Conclusion:**

This scoping review demonstrates that intestinal MNPL research has expanded rapidly but remains methodologically concentrated in simplified in vitro systems and dominated by a limited range of polymer types and particle conditions. Although existing studies have generated important mechanistic insights, future progress will depend on the broader use of physiologically representative intestinal models, environmentally relevant polymers, and more standardized physicochemical characterization protocols. These findings provide a structured evidence map to support more realistic and translationally relevant future studies.

**Clinical trial number:**

Not applicable.

**Supplementary Information:**

The online version contains supplementary material available at https://doi.org/10.1186/s12876-026-05475-0.

## Background

Global plastic production has increased exponentially over the past decades. The widespread use and persistence of plastic materials have resulted in the fragmentation of larger plastic debris into microscopic particles known as microplastics (MPs, 1 μm–5 mm) and nanoplastics (NPs, < 1 μm). These particles are now widely recognized as emerging environmental contaminants and have been detected across diverse environmental compartments including marine ecosystems, freshwater systems, soils, and atmospheric deposition. Due to their small size, persistence, and physicochemical stability, MPs and NPs can disperse widely and interact with biological systems, raising increasing concerns regarding their potential ecological and human health impacts [1, 2].

Human exposure to MPs and NPs occurs through multiple pathways, including ingestion, inhalation, and dermal contact. Among these routes, oral ingestion represents the primary and most direct pathway of environmental exposure to the human body. Microplastic particles have been detected in food products, drinking water, and airborne particulate matter that can be swallowed, indicating that the gastrointestinal tract is a major interface between environmental plastic particles and human physiology. Consistent with this pathway, microplastics have been identified in human stool samples, providing direct evidence of human exposure through the digestive system [3].

The intestinal epithelium functions as a critical biological barrier that regulates nutrient absorption while protecting the organism from potentially harmful substances. This barrier consists of tightly connected epithelial cells, mucus layers, immune components, and interactions with the gut microbiota. Disruption of intestinal barrier integrity may lead to increased epithelial permeability, inflammatory responses, and systemic translocation of foreign particles or toxic compounds. Given their small size and large surface area, MPs and particularly NPs may interact directly with intestinal epithelial cells, potentially triggering oxidative stress, inflammatory signaling, mitochondrial dysfunction, and alterations in epithelial barrier function. Experimental investigations of these interactions have increasingly utilized mammalian models, including human intestinal epithelial cell lines such as Caco-2 and HT-29, intestinal organoids, and rodent models [4].

Beyond the selection of experimental models, the functional consequences of micro- and nanoplastic exposure in intestinal systems are increasingly being documented. A growing body of experimental evidence indicates that exposure to MPs and NPs may induce a variety of biological responses within intestinal systems. These responses include oxidative stress generation, inflammatory signaling activation, mitochondrial dysfunction, apoptosis, and disruption of epithelial tight junctions. Among the various polymer types, polystyrene (PS) particles are the most frequently studied due to their widespread use in experimental models and their well-characterized physicochemical properties. Emerging evidence further suggests that nanoplastics may exhibit enhanced cellular uptake compared with larger microplastic particles, potentially leading to greater intracellular accumulation and biological reactivity [5, 6].

Despite the rapidly increasing number of experimental studies examining the biological effects of MPs and NPs in intestinal systems, the current literature remains highly heterogeneous. Substantial variability exists in polymer composition, particle size distribution, surface characteristics, exposure concentrations, exposure durations, and the biological endpoints evaluated. Furthermore, experimental models range from simple in vitro epithelial systems to complex animal models, often employing markedly different methodological approaches. This lack of methodological standardization complicates the comparison of findings across studies and limits the ability to derive consistent conclusions regarding the toxicological relevance of microplastic exposure. Moreover, current research frequently overlooks the complex interactions between plastic particles and the intestinal mucus layer, a critical yet understudied component of the intestinal barrier that plays a key role in particle trapping and epithelial protection. Importantly, such heterogeneity also constrains the development of robust human health risk assessments for microplastic exposure [7].

Given the diversity of experimental designs and the rapidly expanding nature of the field, a comprehensive mapping of the available evidence is required to better understand current research trends and identify key knowledge gaps. Scoping reviews are particularly well suited for synthesizing evidence in emerging research areas characterized by heterogeneous methodologies and complex experimental frameworks. Unlike traditional systematic reviews that primarily aim to quantify effect sizes, scoping reviews provide a structured evidence map of existing studies, enabling the identification of research clusters, methodological patterns, and priority areas for future investigation. The methodological framework developed by the Joanna Briggs Institute (JBI) and the Preferred Reporting Items for Systematic Reviews and Scoping Reviews (PRISMA-ScR) guidelines provide a standardized approach for conducting and reporting such evidence mapping studies [8].

Several previous reviews have addressed micro- and nanoplastic effects in the gastrointestinal system, but they have generally emphasized specific aspects of the evidence base. Earlier reviews have focused on the human digestive environment and the development of in vitro gut models [9], the interaction of microplastics with the intestinal microbiota [10], foodborne exposure and intestinal health, or broader gastrointestinal disease implications. More recently, a systematic review specifically examined biological effects in human gastrointestinal in vitro models and synthesized 30 studies, with particular emphasis on cellular uptake, oxidative stress, mitochondrial dysfunction, inflammation, and apoptosis [11]. In contrast, the present scoping review maps a broader experimental landscape of mammalian intestinal models and integrates model architecture, polymer identity, particle size, particle condition, exposure scenario, co-exposure, and biological outcome domains across 90 included studies. Rather than focusing primarily on individual toxicological mechanisms or human gastrointestinal cell models, the present review evaluates how methodological concentration within the evidence base may influence the interpretation, comparability, and translational relevance of reported intestinal MNPL effects.

Therefore, this scoping review seeks to systematically map the experimental evidence on the biological effects of micro- and nanoplastics in mammalian intestinal models. By synthesizing available studies across different experimental systems, polymer types, particle characteristics, exposure conditions, and biological endpoints, this review aims to provide a high-level evidence map of the current research landscape and to identify critical gaps that may guide future investigations in microplastic toxicology and environmental health research, with particular emphasis on methodological concentration, particle realism, exposure relevance, and the translational interpretation of intestinal toxicity findings.

## Methods

### Study design

This study was conducted as a scoping review designed to systematically map the experimental evidence on the biological effects of micro- and nanoplastics in mammalian intestinal models. The review followed the methodological framework proposed by the Joanna Briggs Institute (JBI) and was reported in accordance with the Preferred Reporting Items for Systematic Reviews and Scoping Reviews (PRISMA-ScR) guidelines [8].

This scoping review architecture was selected to characterize the methodological landscape of experimental microplastic toxicology studies, a research field currently marked by heterogeneous exposure conditions, variable particle characteristics, and the absence of standardized experimental frameworks. The methodological workflow consisted of five sequential stages:

(i) formulation of research questions, (ii) identification of relevant studies through structured database searches, (iii) study selection using predefined eligibility criteria, (iv) data extraction using a standardized charting framework, and (v) descriptive synthesis and evidence mapping of the included studies.

### Search strategy and information sources

A comprehensive literature search was conducted across three major scientific databases: PubMed, Scopus, and Web of Science Core Collection. These databases were selected because they provide extensive coverage of biomedical, environmental, and toxicological research. The search strategy was designed to capture studies investigating the biological effects of micro- and nanoplastics in intestinal systems. Search terms were developed using combinations of keywords related to microplastics, intestinal models, and toxicological responses. Boolean operators (AND, OR) were applied to combine search terms. Searches were performed within titles, abstracts, and indexing terms, including Medical Subject Headings (MeSH) where applicable in PubMed, in order to maximize retrieval sensitivity. Database-specific search syntaxes were adapted to comply with the indexing structures of each database. In Scopus, the TITLE-ABS-KEY field was used to capture search terms appearing in titles, abstracts, and author keywords. In Web of Science, the TS (Topic) parameter was used to retrieve terms appearing in titles, abstracts, author keywords, and KeyWords Plus. The literature searches were completed on 5 March 2026. The detailed search strategies used for each database are summarized in Table 1.

**Table 1:** Database search strategies used to identify studies on intestinal micro- and nanoplastic toxicity

Database	Search Field	Search Strategy
**PubMed**	Title / Abstract	(microplastic*[tiab] OR micro-plastic*[tiab] OR nanoplastic*[tiab] OR nano-plastic*[tiab] OR “plastic particle*“[tiab] OR “polymer particle*“[tiab] OR “polystyrene microplastic*“[tiab] OR “polyethylene microplastic*“[tiab] OR “polypropylene microplastic*“[tiab]) AND (intestinal[tiab] OR gut[tiab] OR colon[tiab] OR colonic[tiab] OR “intestinal epithelial“[tiab] OR Caco-2[tiab] OR HT-29[tiab]) AND (toxic*[tiab] OR cytotoxic*[tiab] OR “oxidative stress“[tiab] OR ROS[tiab] OR inflammation[tiab] OR permeability[tiab] OR “barrier function“[tiab])
**Scopus**	TITLE-ABS-KEY	TITLE-ABS-KEY (microplastic* OR micro-plastic* OR nanoplastic* OR nano-plastic* OR “plastic particle*” OR “polymer particle*” OR “polystyrene microplastic*” OR “polyethylene microplastic*” OR “polypropylene microplastic*”) AND TITLE-ABS-KEY (intestinal OR gut OR colon OR colonic OR “intestinal epithelial” OR Caco-2 OR HT-29) AND TITLE-ABS-KEY (toxic* OR cytotoxic* OR “oxidative stress” OR ROS OR inflammation OR permeability OR “barrier function”) AND (LIMIT-TO (DOCTYPE, “ar”))
**Web of Science**	Topic (TS)	TS=(microplastic* OR micro-plastic* OR nanoplastic* OR nano-plastic* OR “plastic particle*” OR “polymer particle*” OR “polystyrene microplastic*” OR “polyethylene microplastic*” OR “polypropylene microplastic*”) AND TS=(intestinal OR gut OR colon OR colonic OR “intestinal epithelial” OR “intestinal cell” OR enterocyte OR Caco-2 OR HT-29) AND TS=(toxic* OR cytotoxic* OR “oxidative stress” OR ROS OR inflammation OR permeability OR “barrier function” OR “epithelial barrier”) Refined by: Document Type = Article; Language = English

To minimize the risk of missing relevant studies, the reference lists of all included articles were also manually screened.

### Eligibility criteria

Eligibility criteria were defined using the Population–Concept–Context (PCC) framework commonly applied in scoping reviews. The population included mammalian intestinal systems such as human intestinal epithelial cell lines (e.g., Caco-2, HT-29), primary intestinal epithelial cells, intestinal organoids, and rodent intestinal models. The concept focused on exposure to microplastics or nanoplastics, including commonly investigated polymer types such as polystyrene, polyethylene, polypropylene, polyvinyl chloride, and polyethylene terephthalate. The context was restricted to experimental toxicological studies investigating biological responses following exposure to microplastic or nanoplastic particles. Studies were specifically limited to those generating primary toxicological data, excluding investigations in which plastic particles were used solely as carriers for other compounds (e.g., drug delivery systems) without an independent assessment of the particle’s biological effects.

Studies were included if they met the following criteria:

- original research articles.
- experimental studies conducted in vitro, ex vivo, or in vivo.
- studies investigating exposure to microplastic or nanoplastic particles.
- studies reporting biological or toxicological endpoints.
- articles published in English in peer-reviewed journals.

The following types of publications were excluded:

- review articles, editorials, or commentaries.
- conference abstracts without full text.
- environmental monitoring studies without biological models.
- studies focusing solely on plastic additives (e.g., BPA or phthalates) without particle exposure.
- studies involving non-mammalian organisms such as fish or invertebrates.

The exclusion of non-mammalian organisms was based on the predefined scope of the review, which was specifically focused on mammalian intestinal models to facilitate interpretation of intestinal biological responses within a mammalian physiological context and to maintain consistency with the review question.

### Study selection

Bibliographic records retrieved from the database searches were systematically migrated into Rayyan, a web-based platform designed to support systematic and scoping reviews [12]. Rayyan was used to facilitate duplicate removal and to manage the screening workflow. Title and abstract screening were initially conducted to identify potentially eligible studies according to the predefined criteria. To ensure reviewer independence and minimize selection bias, the initial screening was conducted using Rayyan’s blind mode, allowing reviewers to evaluate records independently without visibility of each other’s decisions. Full-text screening was subsequently performed for studies meeting the inclusion criteria during the first stage. Language eligibility was assessed during the screening process, and only studies published in English were included in the final evidence mapping.

The study selection procedure consisted of two stages:

1. title and abstract screening.
2. full-text eligibility assessment.

Discrepancies between reviewers were resolved through discussion until consensus was reached. The overall selection process is illustrated using a PRISMA-ScR flow diagram.

### Data extraction

Data from the included studies were extracted using a structured data-charting framework developed for this review. The extraction form was designed to capture key study characteristics and experimental parameters relevant to microplastic toxicology. Data extraction was performed independently by both authors (D.Ö.V. and N.V.) for all 90 included studies using the standardized data-charting framework developed for this review. Each author independently extracted information on study characteristics, experimental models, polymer types, particle characteristics, exposure conditions, and biological endpoints. Any discrepancies between the independently extracted datasets were discussed and resolved through consensus between the two authors.

The following information was recorded from each study:

- author and publication year.
- country of origin.
- experimental model (cell line, organoid, or animal model).
- polymer type of the plastic particles.
- particle size and physical characteristics.
- exposure concentration and exposure duration.
- exposure route where applicable.
- experimental assays and analytical methods.
- investigated biological endpoints (e.g., cytotoxicity, oxidative stress, inflammation, epithelial barrier integrity, gene expression).

To account for variability in particle size reporting across studies, plastic particles were categorized into size cohorts to facilitate consistent evidence mapping. Particles were classified as nanoplastics (< 1000 nm) or microplastics (1–5000 μm), enabling comparison of size-dependent biological responses reported in the literature.

### Data synthesis

Given the expected heterogeneity in experimental design, particle characteristics, exposure conditions, and biological endpoints, a quantitative meta-analysis was not considered appropriate. Instead, findings were synthesized using a descriptive evidence-mapping approach. The included studies were categorized according to experimental model type, polymer composition, particle size class, exposure conditions, and biological endpoints investigated. Frequency distributions were calculated to identify the most studied particle types, exposure conditions, and biological responses across the literature. These distributions were subsequently visualized using graphical representations illustrating trends in the literature, including the distribution of experimental models, polymer types, particle size categories, and particle condition categories investigated across studies. In addition to frequency-based mapping, cross-study patterns were examined by comparing biological endpoints across experimental model types, polymer categories, particle size classes, and exposure scenarios. This thematic comparison was used to identify recurring associations and differences in reported biological responses, including oxidative stress and mitochondrial injury, barrier dysfunction, inflammation and immune signaling, cell death and DNA damage, uptake and translocation, co-exposure effects, and gut microbiota-related responses. Because of the heterogeneity of the included studies, these comparisons were interpreted as evidence-mapping patterns rather than pooled estimates of effect. A schematic overview of the synthesized findings was prepared using ChatGPT (OpenAI), and its scientific content was critically reviewed and verified by both authors against the included literature.

## Results

### Study selection

The systematic literature search conducted across PubMed, Scopus, and Web of Science yielded a total of 3,540 records, including 1,089 records from PubMed, 1,223 from Scopus, and 1,228 from Web of Science. Following the import of bibliographic records into the Rayyan platform, duplicate entries were identified and removed. After duplicate removal, 1,616 unique records remained for title and abstract screening.

**Fig. 1:**
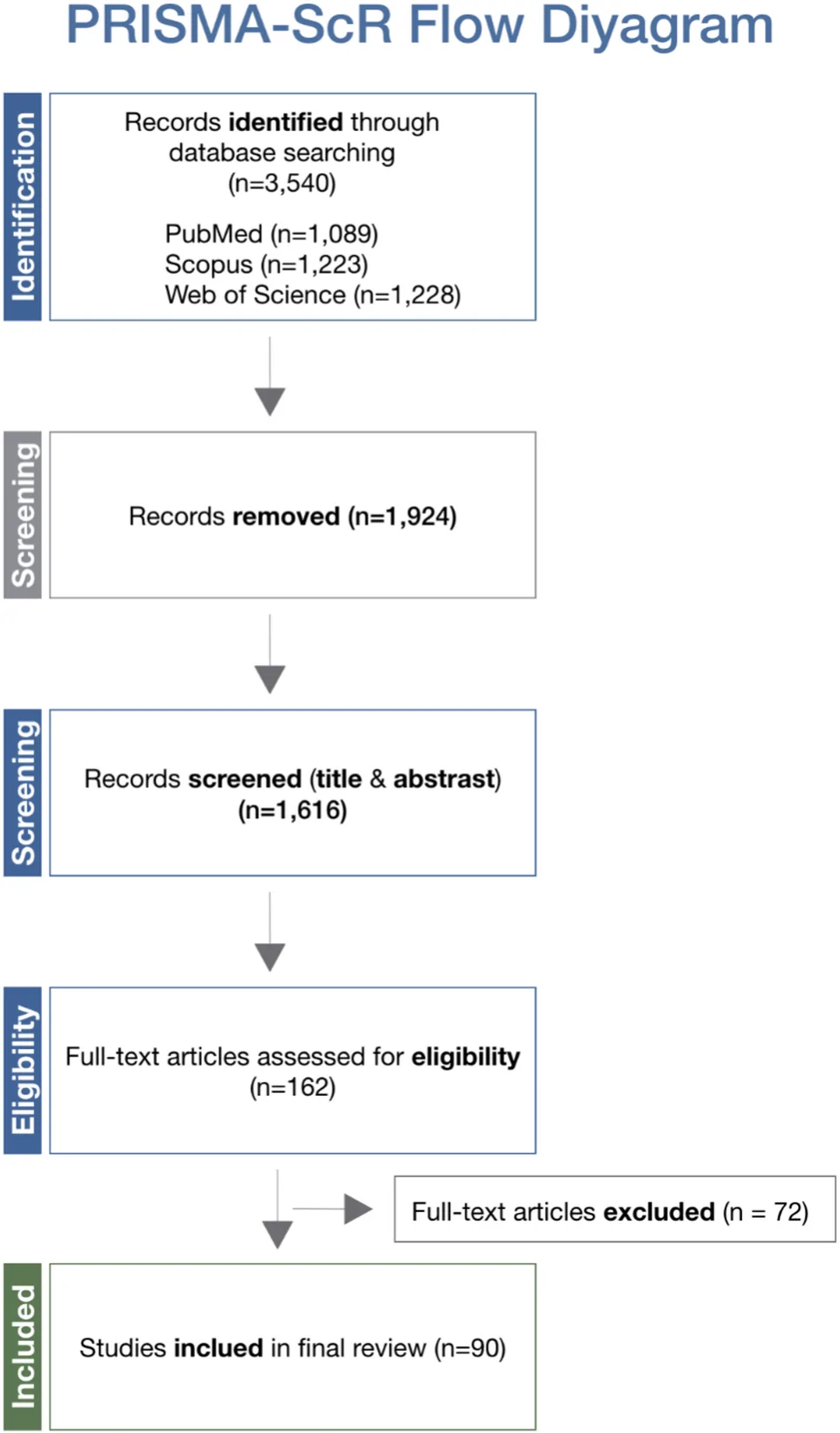
PRISMA-ScR flow diagram illustrating the study selection process. Records were identified through searches of PubMed, Scopus, and Web of Science (*n* = 3,540). After removal of duplicate records (*n* = 1,924), 1,616 records were screened by title and abstract, of which 1,454 were excluded. A total of 162 full-text articles were assessed for eligibility, and 72 were excluded following full-text assessment. Ultimately, 90 studies met the predefined eligibility criteria and were included in the final evidence mapping

During the first screening stage, 1,454 records were excluded because they did not meet the predefined eligibility criteria, primarily due to the absence of experimental exposure to micro- or nanoplastic particles, the use of non-intestinal biological models, or the investigation of plastic additives rather than plastic particles themselves. The remaining 162 articles were subsequently subjected to full-text assessment. Full-text screening resulted in the exclusion of 72 studies for reasons including lack of primary toxicological data, use of non-mammalian biological models, or insufficient methodological information regarding particle exposure. Ultimately, 90 studies met the eligibility criteria and were included in the final evidence mapping. The complete study selection process is summarized in the PRISMA-ScR flow diagram (Fig. 1).

### Characteristics of included studies

The 90 included studies collectively delineate a rapidly evolving evidence base on the intestinal toxicology of micro- and nanoplastics in mammalian systems. The publication pattern indicates a clear acceleration of research activity in recent years, underscoring growing recognition of the gastrointestinal tract as a primary and biologically meaningful interface of plastic particle exposure. Across the literature, in vitro models clearly predominate, with Caco-2-based systems serving as the principal experimental platform for interrogating epithelial barrier injury, cellular uptake, and downstream stress responses. A subset of studies moved beyond conventional monoculture designs by incorporating mucus-producing co-cultures, thereby improving physiological relevance. In vivo rodent studies complemented this evidence by capturing higher order responses, including tissue injury, inflammatory signaling, microbial perturbation, and systemic consequences of intestinal exposure. Nevertheless, the limited use of organoids, tri-culture models, and gut-on-a-chip systems suggests that the field remains methodologically weighted toward reductionist designs, despite an emerging shift toward more complex and translationally informative models. To identify the studies that most strongly shaped the mechanistic and translational interpretation of the field, we synthesized the major key studies in Table 2. To further organize the evidence base beyond representative key studies, the included literature was thematically synthesized according to experimental focus, exposure scenario, and dominant biological outcome patterns, as presented in Table 3. In addition, cross-study comparisons were used to examine recurring relationships between experimental models, polymer categories, particle size classes, exposure scenarios, and biological outcomes. This synthesis was used to distinguish recurring evidence patterns from findings that remained limited to individual experimental systems.

**Table 2:** Major key studies highlighting the core mechanisms and advanced models of intestinal MNPL toxicity

Ref.	Experimental Model	Polymer Particle Type	Particle Size	Exposure Scenario	Main Endpoints	Core Mechanistic Contribution
[13]	Caco-2, HepaRG	PLA, PMMA, MF	25 nm–2 μm	Standard in vitro	Uptake, subcellular detection, viability	Demonstrated subcellular detection and oral bioavailability patterns of food-relevant submicron/nanoplastics.
[14]	3D co-culture (M-cell, mucus, intestinal–liver)	PLA, PMMA, MF	25 nm–2 μm	Physiological barrier models	Uptake, transport, hepatic transfer	Highlighted M-cell- and mucus-dependent uptake differences and downstream hepatic transfer.
[15]	Caco-2	PLA, PMMA, MF	25 nm–2 μm	Artificial digestion	Aggregation, uptake, transport, viability	Demonstrated that digestive protein corona formation modifies particle interaction and toxicity.
[16]	Caco-2, HepG2	PE, PP, PET, PVC	Variable	Polymer diversity	Particle uptake, cytotoxicity	Resolved polymer bias by comparing major environmentally relevant plastics.
[17]	In vitro gut/liver models	Microplastics Nanoplastics	Variable	Dispersant variation	Toxicity artefacts, uptake, cytotoxicity	Revealed methodological flaws caused by dispersant-driven apparent toxicity.
[18]	Gut–liver-on-a-chip	PS	100 nm	Peristaltic flow	Transport, hepatic uptake, ROS, organ crosstalk	Demonstrated the critical role of intestinal peristalsis in mitigating downstream liver damage.
[19]	Caco-2	PP, PS	Micro/nano range	Real takeaway containers (freeze/microwave)	Viability, uptake, lysosomal alterations, ROS	Established thermal release of MPs from real-world food containers and linked them to subcellular intestinal stress.
[20]	RKO, HT-29, HCT-116, HIEC-6, mice	PS	100 nm	Multi-model exposure	Growth/survival, intracellular accumulation, autophagic flux	Identified impaired autophagic flux as a major intestinal nanoplastic toxicity mechanism across normal and tumor-derived intestinal cells.
[21]	Caco-2 human intestinal cells	PP	Irregular product-derived MPs	Real baby bottle exposure scenario	ROS, lipid peroxidation, IL-6, TNF-α, inflammasome signaling	Linked infant feeding bottle-derived MPs to intestinal inflammation through the ROS/NLRP3/Caspase-1/IL-1β axis.
[22]	In vivo (mice, DSS-induced UC model)	PET	5 μm	Ulcerative colitis disease model	Multi-omics, hub genes, WB validation	Demonstrated that PET-MPs aggravate pre-existing colitis and provided disease-context mechanistic evidence.
[23]	Human intestinal epithelial co-culture	PET	Diet-relevant PET-MPs	Digested foodborne exposure	Cytotoxicity, oxidative stress, barrier dysfunction, AMPK, DNA damage	Discovered the mitochondrial–AMPK–DNA damage pathway as a central mechanism of PET-MP intestinal injury.
[24]	Human intestinal co-culture / composite food-digesta model	PET	Predominantly 20–100 μm fraction	Digested foodborne exposure	Mitochondrial respiration, oxidative stress, metabolic profiling	Detailed the hyperactivation of mitochondrial Complex I as a driver of intestinal bioenergetic collapse.
[25]	Caco-2 intestinal barrier / Transwell model	PET	Microscale PET-MPs	Co-exposure with PFOA	TEER, tight junctions, ROS, MMP, LDH	Showed synergistic barrier disruption and tight-junction injury during PET-MP/PFOA co-exposure.
[26]	3D tri-culture small intestinal model	PS	26 nm	Digestion and uptake	Translocation, TEER, LDH, ROS, mechanistic inhibition	Validated both passive and active uptake mechanisms, including phagocytosis/endocytosis, in a physiologically relevant triculture model.

**Table 3:** Thematic synthesis of the 90 included studies across experimental models, exposure scenarios, and biological outcomes

Evidence Theme	Experimental Focus	Typical Models Used	Typical Exposure Material Pattern	Main Biological Outcomes	No. of Studies*	Representative Studies
**Uptake and translocation**	Cellular internalization, epithelial passage, particle fate, translocation across intestinal barriers	Caco-2, Transwell systems, tri-culture models, intestinal–liver platforms	PS, PET, PLA, PMMA, MF; virgin or digested particles; size-dependent uptake designs	Internalization, transport, translocation, intracellular localization, hepatic transfer	21	[13], [14], [26]
**Barrier dysfunction and permeability**	Disruption of epithelial integrity and tight-junction architecture	Caco-2, Caco-2/HT-29, Transwell barrier systems, intestinal co-cultures	PS, PET, PP; single exposure and co-exposure settings	TEER reduction, increased permeability, tight-junction loss, mucus-layer alterations	19	[23], [25], [27]
**Oxidative stress and mitochondrial injury**	Redox imbalance, mitochondrial dysfunction, metabolic injury	Caco-2, HIEC-6, co-cultures, rodent intestine	PS, PET, aged/weathered particles, digested particles	ROS generation, lipid peroxidation, MMP loss, ATP depletion, bioenergetic dysfunction	27	[19], [23], [24]
**Inflammation and immune signaling**	Cytokine dysregulation, inflammasome activation, inflammatory amplification	Caco-2, rodent models, disease-context intestinal models	PS, PP, PET; product-derived particles; disease-context exposure	IL-6, IL-8, TNF-α, inflammasome activation, immune perturbation	11	[21], [22]
**Cell death**,** DNA damage**,** and autophagy**	Apoptosis, genotoxicity, autophagic flux impairment, proliferation arrest	Caco-2, HIEC-6, HT-29, intestinal co-cultures, mice	PS, PET, nanoscale particles, digested particles	Apoptosis, DNA damage, autophagy disruption, proliferation arrest, genotoxic stress	17	[20], [23], [28]
**Co-exposure and vector effects**	Interactions between MNPLs and other pollutants, toxins, or co-contaminants	Caco-2, Transwell systems, rodent/disease models	PET + PFOA, PS + NOR, MNPLs + metals/pesticides	Synergistic cytotoxicity, barrier aggravation, enhanced oxidative or inflammatory responses	18	[25], [29], [30]
**Digested**,** product-released**,** or environmentally transformed particles**	More realistic exposure scenarios beyond pristine commercial particles	Simulated digestion models, product-derived exposure models, Caco-2, co-cultures	Digested PET-MPs, product-released PP/PS MPs, aged/weathered PS	Altered uptake, enhanced toxicity, consumer-relevant intestinal stress, transformation-dependent reactivity	28	[19], [24], [31]
**Multicellular and advanced intestinal models**	Higher physiological complexity, organ crosstalk, translational modeling	Tri-culture systems, organoids, gut-on-a-chip, gut–liver-on-a-chip, 3D intestine barriers	Mixed polymers, digested particles, dynamic flow systems	More realistic transport behavior, organ crosstalk, barrier modulation, mechanistic refinement	18	[14], [18], [26]
**Gut microbiota and probiotic interactions**	Effects of MNPL exposure on gut microbial composition, microbial functions, and probiotic-associated responses	Gut microbiota models, fecal microbiome systems, Caco-2–microbiota models, and probiotic-associated intestinal models	PE, PET, PS, PP, PVC and mixed/product-derived particles	Gut dysbiosis, altered microbial composition or diversity, microbial functional changes, probiotic viability or modulation of particle-associated effects	9	[32], [33], [34]

* Theme-based occurrence counting was applied; therefore, a single study could contribute to more than one thematic category

The thematic mapping also identified a comparatively smaller but distinct body of evidence addressing interactions between MNPLs and the gut microbiota or probiotic systems. Nine studies investigated microbiota composition, microbial diversity or function, probiotic viability, or probiotic-mediated modulation of particle-associated intestinal responses. These studies included human-derived fecal or simulated microbiota systems, intestinal epithelial–microbiota models, and probiotic-associated experimental configurations, indicating that microbiota-related effects are emerging but remain less extensively investigated than epithelial barrier, oxidative stress, and cellular injury endpoints.

### Landscape and complexity of experimental models

The mapping of experimental architectures reveals a methodological hierarchy characterized by a trade-off between physiological complexity and experimental scalability. Simplified two-dimensional (2D) monoculture systems represent a major component of the literature, with Caco-2 epithelial monolayers serving as the primary platform for investigating intestinal responses to micro- and nanoplastic exposure. The widespread adoption of this model is largely driven by its high-throughput capacity and well-established protocols for assessing epithelial transport processes, cytotoxicity, and oxidative stress responses.

Beyond monoculture systems, a substantial proportion of studies employed multicellular co-culture models, most commonly combining Caco-2 and HT-29 cells. These models introduce mucus-secreting goblet-like cells into the experimental architecture and therefore provide a closer approximation of the intestinal mucosal environment compared with standard epithelial monolayers. The presence of a mucus layer is particularly relevant in microplastic research, as it influences particle–epithelium interaction dynamics.

A considerable portion of the literature also relies on in vivo rodent studies, which provide an organism-level perspective that cannot be replicated by in vitro systems. Experimental work conducted primarily in C57BL/6 mice and Sprague–Dawley rats has enabled investigation of more complex endpoints, including intestinal inflammation, alterations in gut microbiota composition, and systemic distribution of ingested plastic particles.

**Fig. 2:**
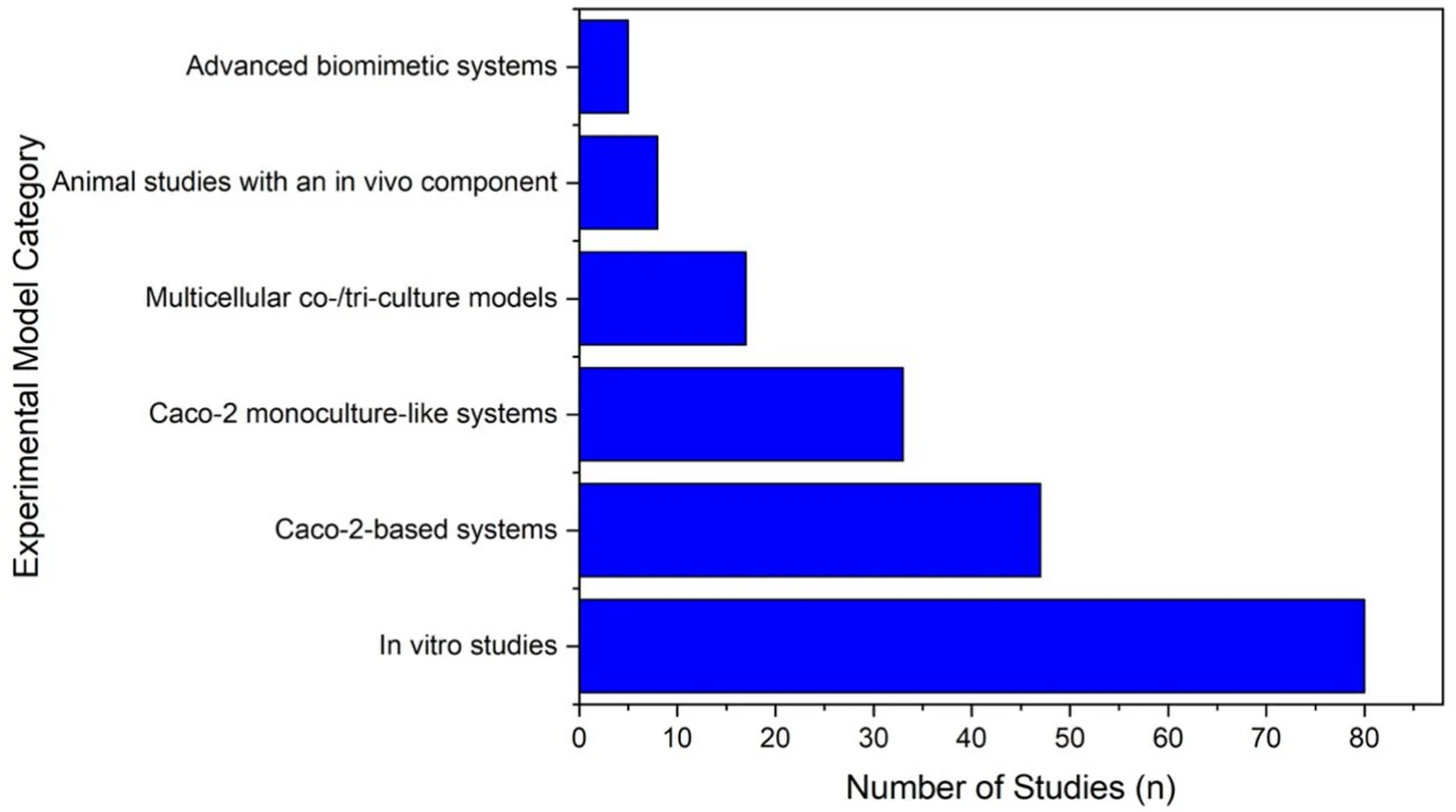
Experimental models across 90 included studies. Occurrence counting was applied; therefore, categories are not mutually exclusive. In vitro studies predominated (*n* = 80), followed by Caco-2-based systems (*n* = 47) and Caco-2 monoculture-like models (*n* = 33). Multicellular co-/tri-culture models (*n* = 17), animal studies with an in vivo component (*n* = 8), and advanced biomimetic systems including organoids and chip-based platforms (*n* = 5) were less frequently represented

In contrast, advanced three-dimensional experimental platforms, such as intestinal organoids and microfluidic “gut-on-a-chip” systems, remain rarely represented in the current literature. The overall distribution of experimental models across the included studies is illustrated in Fig. 2.

### Physicochemical diversity and polymer bias

**Fig. 3:**
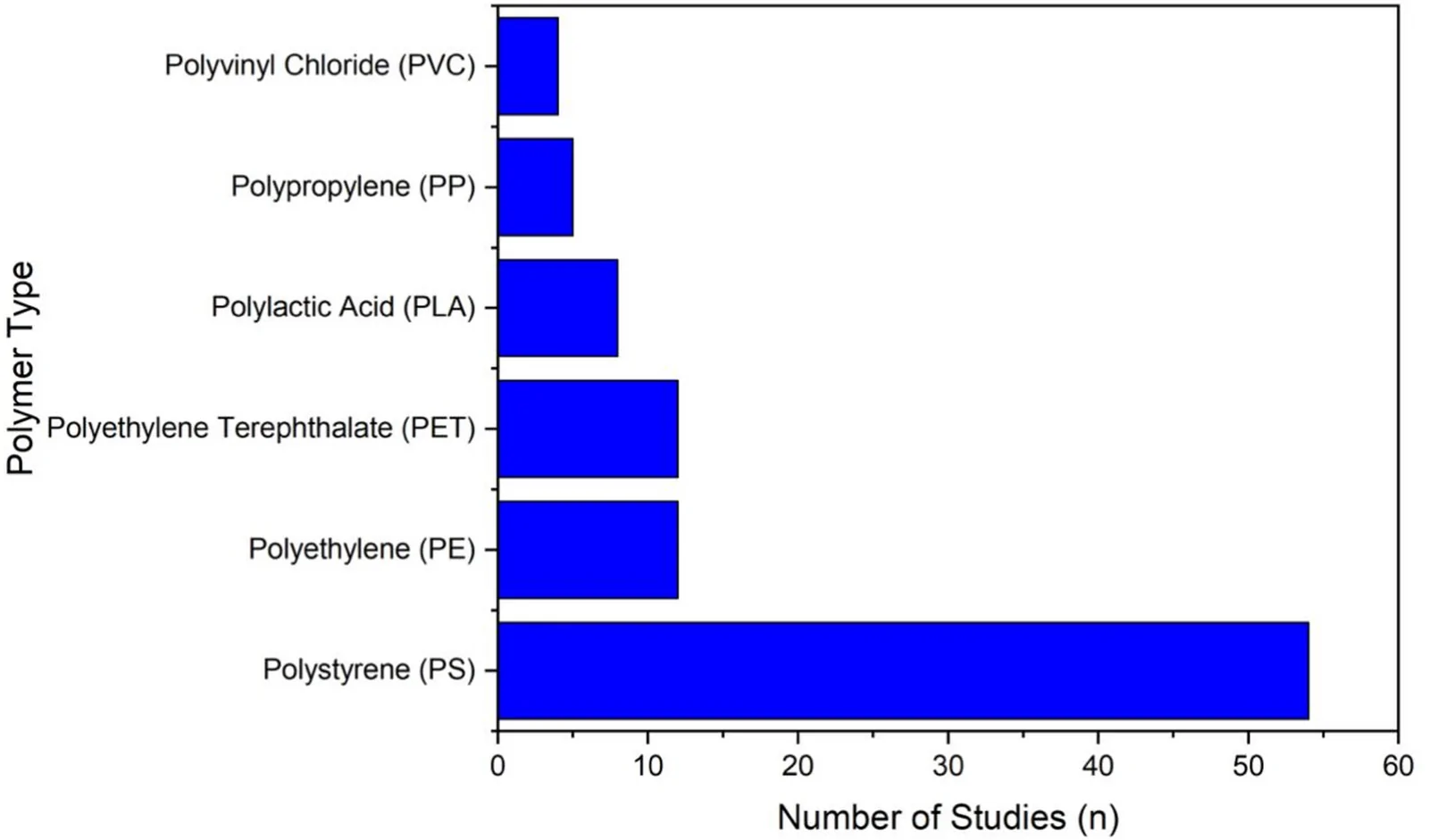
Distribution of polymer types investigated across the 90 included studies. Occurrence counting was applied; therefore, studies investigating more than one polymer type were counted in each relevant category. Polystyrene dominated the evidence base (*n* = 54, 60.0%), whereas polyethylene and polyethylene terephthalate were each represented in 12 studies (13.3%). Polypropylene, polyvinyl chloride, and polylactic acid were investigated less frequently

The physicochemical landscape of particles investigated across the included studies reveals substantial heterogeneity in polymer composition, particle size, and particle condition. A clear polymer bias was evident, with polystyrene (PS) representing the most frequently investigated material. The predominance of PS may be partly attributable to its commercial availability as relatively uniform particles and its well-characterized properties in experimental systems. In contrast, environmentally prevalent polymers such as polyethylene (PE), polypropylene (PP), and polyethylene terephthalate (PET) were comparatively underrepresented. This discrepancy highlights a methodological tension between laboratory convenience and environmental relevance. The distribution of polymer types investigated across the included studies is illustrated in Fig. 3.

**Fig. 4:**
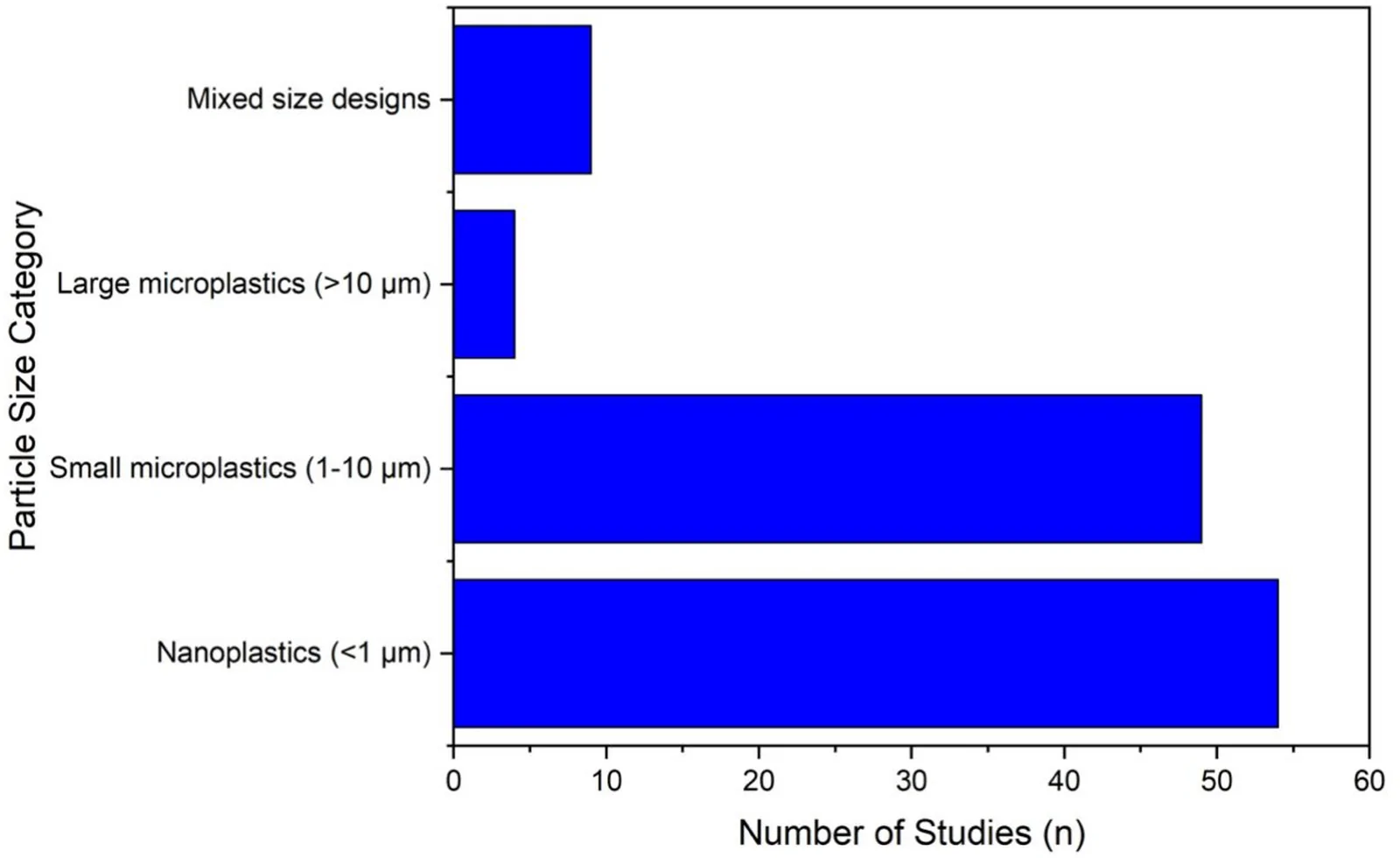
Distribution of particle size categories investigated across the 90 included studies. Occurrence counting was applied; therefore, studies examining more than one particle size class were counted in each relevant category. Nanoplastics (< 1 μm) represented the most frequently investigated size category (*n* = 54, 60.0%), followed by small microplastics (1–10 μm; *n* = 49, 54.4%). Large microplastics (> 10 μm) were examined less frequently (*n* = 4, 4.4%), while a smaller subset of studies used mixed size designs spanning more than one particle class (*n* = 9, 10.0%)

Particle size represents another important determinant of biological interaction. Both microplastics and nanoplastics were investigated, with reported particle dimensions ranging from the nanoscale to larger microscale fractions. Nanoplastics were frequently employed in mechanistic studies, reflecting their suitability for investigating cellular uptake, intracellular localization, and particle–cell interactions. The distribution of particle size categories across the included studies is presented in Fig. 4.

**Fig. 5:**
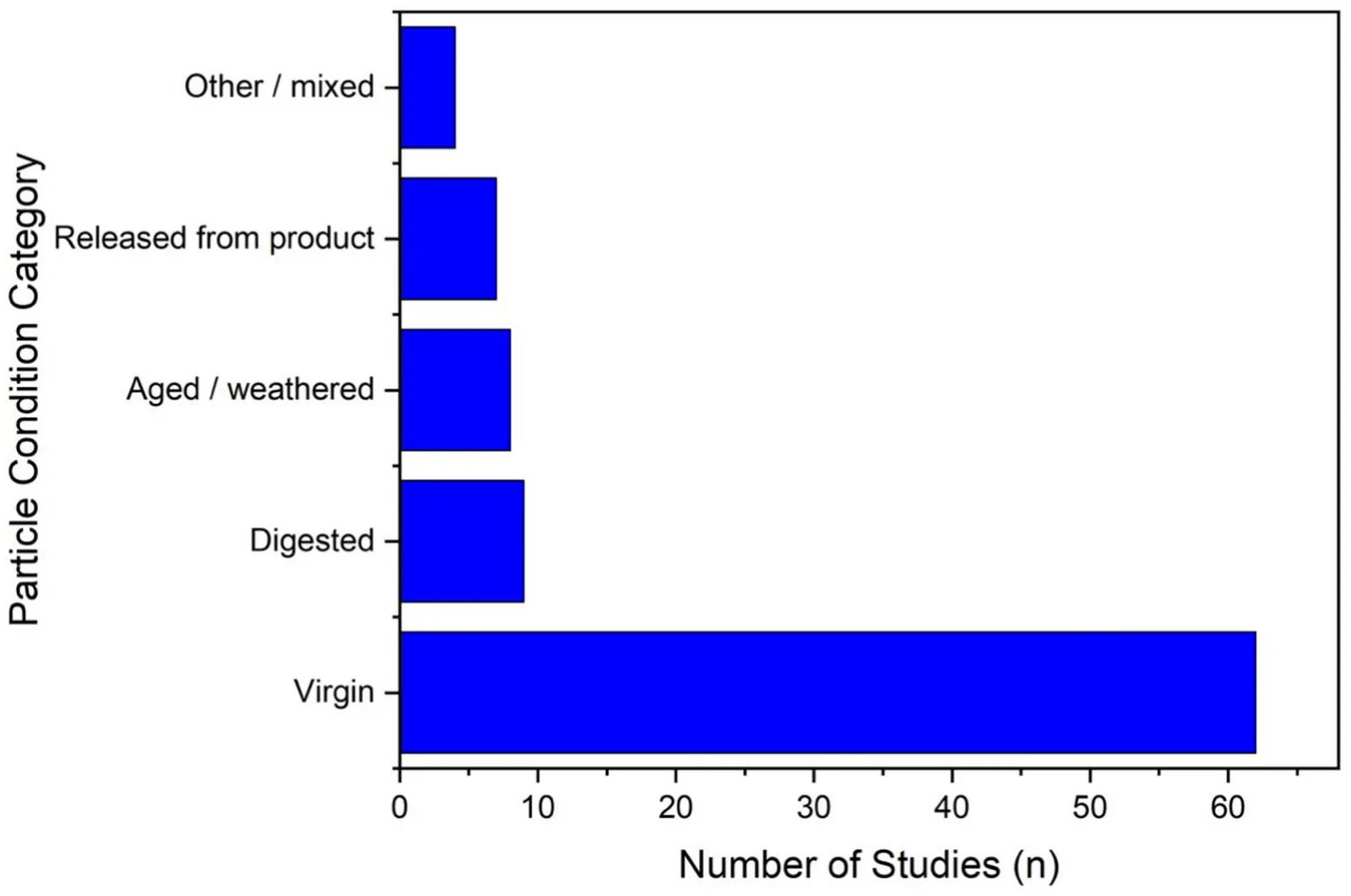
Distribution of particle condition categories across the 90 included studies. Studies were classified into mutually exclusive categories according to particle condition as reported in the final evidence-charting table. Most studies used virgin particles (*n* = 62, 68.9%), whereas digestion-modified (*n* = 9, 10.0%), aged or weathered (*n* = 8, 8.9%), and product-released particles (*n* = 7, 7.8%) were less frequently represented. A small number of studies were grouped as other or mixed conditions (*n* = 4, 4.4%)

Particle condition further contributes to variability in experimental outcomes. Most included studies relied on virgin particles, whereas fewer incorporated digestion-modified, aged or weathered, or product-released materials. These transformation states are relevant because they can alter surface roughness, functional group composition, biomolecular adsorption, aggregation behavior, and biological reactivity within the intestinal microenvironment. The distribution of particle condition categories across the included studies is summarized in Fig. 5. Studies were classified into mutually exclusive categories according to particle condition as reported in the final evidence-charting table.

Overall, the evidence map demonstrates substantial heterogeneity in the physicochemical characteristics of particles investigated across studies. The predominance of PS, nanoscale particles, and virgin materials indicates that the current experimental literature is concentrated around relatively standardized laboratory particles rather than the broader diversity of particles encountered in environmental and consumer-related exposure scenarios. This pattern reinforces the need for more consistent physicochemical characterization and greater incorporation of environmentally and physiologically transformed particles in future intestinal MNPL research.

**Fig. 6:**
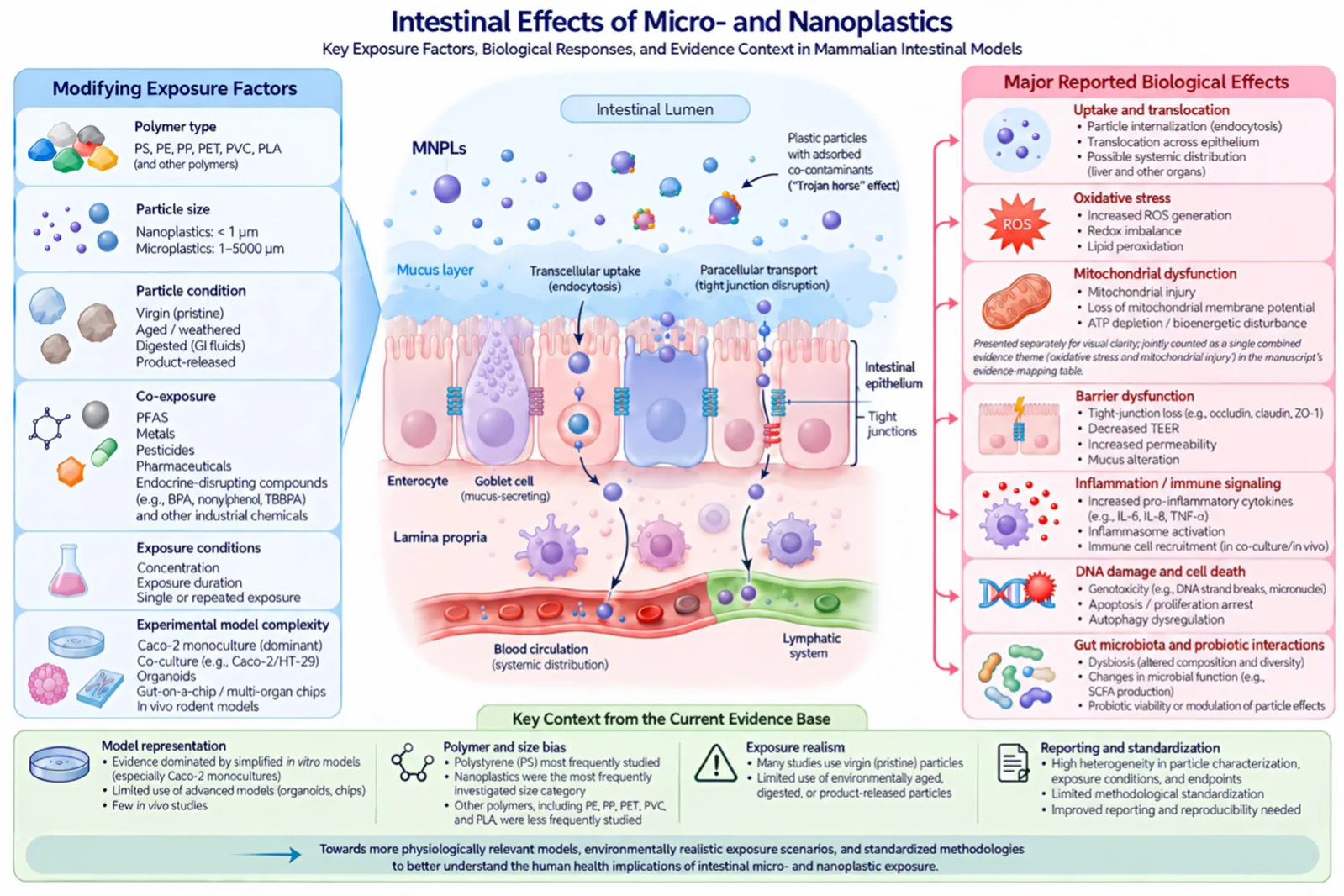
Graphical summary of the major biological effects and modifying factors associated with intestinal micro- and nanoplastic exposure in mammalian intestinal models. The schematic integrates the major biological response domains identified across the included studies, including uptake and translocation, oxidative stress and mitochondrial dysfunction, barrier impairment, inflammation and immune signaling, DNA damage and cell death-related responses, and gut microbiota and probiotic interactions. The reported responses may be influenced by particle size, polymer type, particle condition, co-exposure context, exposure conditions, and experimental model complexity. The illustration is a schematic representation and is not anatomically to scale. The illustration was generated using ChatGPT (OpenAI); see the AI Declaration for further details

An integrated graphical synthesis of the major biological responses associated with intestinal MNPL exposure, together with the principal exposure-related and methodological factors influencing their interpretation, is presented in Fig. 6.

## Discussion

### Methodological monoculture: the dominance of simplified epithelial models

A defining characteristic of the current research landscape is the strong reliance on simplified in vitro experimental architectures. Caco-2-based systems constituted the dominant experimental platform in the evidence base, with monoculture-like configurations representing a substantial proportion of included studies [13, 15, 16]. This methodological concentration is important because model architecture can influence both the magnitude and the biological dimension of responses observed following MNPL exposure.

The widespread adoption of Caco-2 monocultures is technically justified by their ability to differentiate into polarized enterocyte-like monolayers and provide a robust and reproducible platform for assessing epithelial barrier integrity, transepithelial transport, and cytotoxic responses. However, this convenience introduces a notable “complexity gap.” Standard Caco-2 monocultures lack a functional mucus layer produced by goblet cells, although ingested particles must interact with the intestinal mucus barrier before reaching epithelial surfaces. Consequently, simplified epithelial systems may overrepresent direct particle–cell interactions while underrepresenting mucus-mediated particle trapping and modulation of epithelial exposure.

The emergence of multicellular co-culture and tri-culture models represents an important step toward addressing these limitations. By incorporating mucus-secreting cells, immune-associated components, or more complex epithelial configurations, these systems provide a more physiologically relevant representation of the intestinal mucosal interface and allow investigation of mucus-dependent transport, particle bioavailability, and interactions between different intestinal cell populations. Advanced co-culture and tri-culture studies have demonstrated that model architecture can influence particle transport, uptake, and downstream cellular responses [14, 26]. Similarly, animal models extend the outcome spectrum to tissue-level inflammation, microbiota alterations, and systemic consequences, whereas organoid and gut-on-a-chip approaches provide opportunities to examine more complex barrier behavior and dynamic exposure conditions. Thus, differences in reported toxicity across studies should be interpreted in the context of model architecture rather than attributed solely to intrinsic differences in particle toxicity.

Despite these developments, higher-order biological complexity remains underrepresented. Advanced biomimetic systems, including intestinal organoids and chip-based platforms, are still uncommon in the evidence base. Their broader adoption is constrained by technical complexity, cost, and limited standardization, although emerging gut-on-a-chip studies have demonstrated the added value of dynamic flow, peristaltic motion, and gut–liver crosstalk for interpreting MNPL transport and downstream tissue injury [18]. In vivo studies similarly provide essential organism-level information but remain subject to interspecies differences in gastrointestinal physiology, including intestinal morphology, pH gradients, and microbiota composition. Disease-context and multi-model studies are particularly valuable because they extend the evidence beyond simplified epithelial endpoints toward organism-level biological consequences [20, 22].

Taken together, the current evidence suggests that simplified epithelial systems remain valuable for mechanistic investigation but provide only a partial representation of the intestinal microenvironment. Greater integration of mucus-, microbiota-, immune-, and flow-inclusive models will therefore be important for determining which MNPL responses observed in simplified systems remain relevant under more physiologically representative conditions.

### Quality, reporting, and reproducibility of the included evidence

Although formal risk-of-bias assessment was not undertaken, as the primary objective of this scoping review was to map the breadth and characteristics of the available evidence, the methodological and reporting characteristics of the included studies provide important information about the interpretability and reproducibility of the current evidence base. Across the 90 included studies, substantial variation was observed in experimental models, particle characteristics, exposure concentrations, exposure durations, and biological endpoints. This heterogeneity does not necessarily indicate poor study quality; rather, it limits the extent to which findings from individual studies can be directly compared or generalized across experimental systems.

A particularly important concern relates to the reproducibility of exposure characterization. Particle size, morphology, exposure concentration, and particle condition were not reported with comparable levels of detail across studies. In many cases, particle size was provided only as a broad category rather than as a precise numerical value or size distribution, while exposure concentrations and experimental conditions also varied substantially. Differences in particle preparation, dispersion procedures, characterization methods, exposure media, and exposure duration may further influence particle aggregation, sedimentation, cellular availability, and effective exposure at the intestinal interface. Consequently, differences in reported biological responses may reflect not only intrinsic differences between polymers or particle sizes but also variation in experimental design and exposure configuration. This is particularly relevant when comparing pristine commercial particles with aged, digested, or product-derived materials, which may differ substantially in their physicochemical and biological behavior.

Future studies would benefit from more consistent reporting of particle identity and physicochemical characteristics, including polymer composition, particle size distribution, morphology, surface characteristics, particle condition, preparation procedures, exposure concentration, and exposure duration. Standardized reporting of these parameters, together with appropriate controls and clearly defined exposure protocols, would improve reproducibility and facilitate more meaningful cross-study comparisons. Such improvements would also strengthen the interpretation of dose–response relationships by allowing biological effects to be considered in relation to the complete exposure configuration rather than concentration alone. These reporting improvements should complement rather than replace physiologically representative intestinal models, as methodological rigor and biological relevance are both necessary for strengthening the evidence base for intestinal MNPL toxicity.

### Polymer bias and environmental disconnect

Another important pattern emerging from the evidence map is the pronounced polymer bias observed in experimental intestinal toxicology studies. PS clearly dominates the current evidence base, whereas environmentally prevalent polymers such as PE, PP, and PET are comparatively underrepresented. This imbalance is particularly notable given that studies directly comparing major environmentally relevant polymers have shown that intestinal uptake and cellular responses may differ across PE, PP, PET, and PVC particles, thereby challenging a PS-centered interpretation of intestinal MNPL toxicity [16]. Taken together, the available evidence suggests that polymer identity may contribute to differences in intestinal particle uptake and cellular responses; however, the current evidence base does not permit a robust polymer-specific ranking of toxicity. The greater number of reported biological effects associated with PS should therefore be interpreted in the context of study availability and experimental representation rather than as evidence that PS is intrinsically more toxic than other polymers. This polymer imbalance represents an important source of uncertainty when extrapolating findings from experimental models to environmentally relevant mixed-polymer exposures.

The dominance of PS is likely driven in part by experimental pragmatism. Commercially available PS particles are typically produced with relatively uniform size distributions and well-characterized physicochemical properties, making them suitable for controlled mechanistic studies of epithelial transport, cellular uptake, oxidative stress, and inflammatory responses. At the same time, methodological work has shown that apparent particle-related effects may also be influenced by dispersants and other preparation-related variables, underscoring how experimental convenience can shape not only polymer choice but also toxicological interpretation [17].

However, this strong reliance on PS introduces an important limitation regarding the environmental representativeness of current experimental systems. Environmentally encountered plastic particles comprise a broader diversity of polymers, shapes, surface properties, and transformation states than is typically represented by commercially prepared PS particles. The comparatively limited representation of PE, PP, PET, and other environmentally relevant materials therefore creates a disconnect between laboratory model particles and realistic exposure scenarios. This gap is only partially addressed by studies using product-released or digestion-modified particles, including particles generated from food containers, infant feeding bottles, or foodborne PET exposure models, which more closely approximate real-world intestinal exposure conditions [19, 21, 23, 24].

Environmental relevance also extends beyond polymer identity to the chemical context in which MNPLs are encountered. The thematic mapping identified studies examining co-exposure or vector-related effects involving contaminants such as PFOA, PFOS, norfloxacin, nonylphenol, tetrabromobisphenol A, silver-based materials, arsenic, and boscalid. The available evidence does not indicate a single uniform interaction pattern; rather, several studies reported enhanced cellular or barrier-related effects under combined exposure conditions. For example, PET microplastics combined with PFOA were associated with synergistic barrier disruption and tight-junction injury in a Caco-2 intestinal barrier model, whereas aged polystyrene nanoplastics enhanced the combined toxic effects observed with norfloxacin. These findings indicate that MNPL toxicity may depend not only on intrinsic particle characteristics but also on the chemical environment surrounding the particle.

The potential mechanisms underlying these combined effects are likely to be multifactorial. MNPLs may adsorb or transport co-contaminants, alter their local availability at the intestinal interface, or modify particle uptake and cellular internalization. Conversely, co-contaminants may alter particle surface properties, cellular susceptibility, or barrier integrity and thereby modify the biological response to MNPLs. However, because the included studies used different polymers, particle sizes, co-contaminants, exposure sequences, concentrations, and intestinal models, the current evidence is insufficient to establish a consistent quantitative relationship between particle–contaminant combinations and toxicity. The principal evidence gap is therefore not the absence of combined-exposure research, but the lack of standardized experimental designs capable of distinguishing synergistic, additive, or antagonistic interactions across studies.

A further environmental disconnect arises from the physical state of the particles used experimentally. The evidence base was strongly weighted toward virgin particles, whereas aged or weathered, product-released, and digestion-modified materials were less frequently investigated. This imbalance is important because environmental and gastrointestinal transformation processes can alter particle surface chemistry, roughness, aggregation, biomolecular adsorption, and biological reactivity. Studies using transformed particles suggest that these altered exposure states may modify uptake behavior, barrier responses, oxidative injury, and downstream cellular stress, indicating that transformation history is not merely a contextual characteristic but a biologically consequential variable [19, 23, 24].

Most experimental studies use relatively uniform particles that permit precise control of dose–response relationships. In contrast, environmental microplastics commonly exhibit heterogeneous shapes, surface topographies, and chemical compositions. Such structural variability may influence particle transport through mucus, cellular uptake, membrane interactions, and the formation and composition of the protein corona at the particle–biological interface. Consequently, findings obtained using highly standardized laboratory particles should not automatically be generalized to the broader spectrum of particles encountered in environmental and consumer-related exposure scenarios.

Taken together, the evidence indicates that the environmental disconnect in intestinal MNPL toxicology is multidimensional, involving not only polymer selection but also particle transformation, physical characteristics, and co-exposure context. Future studies should therefore move beyond single-polymer, pristine particle systems and incorporate environmentally prevalent polymers, aged or weathered materials, product-released or digestion-modified particles, and relevant co-contaminants where appropriate. Such approaches would improve the environmental representativeness of experimental models while allowing the biological consequences of particle characteristics and exposure context to be evaluated more systematically.

### The “Size Gap”: mechanistic granularity vs. environmental scale

Particle size represents one of the most important biophysical determinants of micro- and nanoplastic (MNPL) interactions within the gastrointestinal environment. As illustrated in Fig. 4, the current evidence base demonstrates a pronounced nano-centric orientation. Nanoplastics were the most frequently investigated size category, whereas larger microplastic fractions were comparatively underrepresented. Because some studies examined more than one particle size category, these occurrence-based distributions should be interpreted as patterns of research emphasis rather than mutually exclusive study counts. The predominance of nanoplastics in the evidence base should not, however, be interpreted as evidence of inherently greater toxicity compared with microplastics. Rather, it reflects the strong representation of nanoscale particles in mechanistic and cellular studies, where their small size facilitates investigation of uptake, intracellular localization, and particle–cell interactions. Direct comparisons between size classes remain relatively limited and are often influenced by differences in polymer type, surface characteristics, exposure concentration, and experimental model. Accordingly, the observed nano-centric pattern primarily indicates a research and methodological emphasis rather than a definitive size-dependent toxicity hierarchy.

The predominance of nanoplastics in experimental systems is largely explained by their physicochemical properties. Due to their extremely small size and high surface area-to-volume ratio, nanoplastics exhibit enhanced interfacial interactions with epithelial cell membranes. Consequently, nanoscale particles are frequently used in mechanistic studies investigating endocytic uptake pathways, translocation behavior, and intracellular localization, as illustrated by advanced uptake-oriented studies using intestinal barrier and tri-culture platforms [13, 15, 26]. These designs have been particularly informative for clarifying how particle size shapes epithelial internalization and downstream transport across the intestinal interface.

However, the strong emphasis on nanoscale particles introduces an important scale imbalance between experimental models and environmentally relevant exposure scenarios. Although nanoplastics may emerge as secondary degradation products of larger plastic debris, real-world exposure is likely to involve a broader distribution of particle sizes, particularly micro-scale fragments generated through physical fragmentation processes. Comparative studies using multiple size classes have shown that intestinal uptake, interaction intensity, and toxicodynamic profiles can differ across nano- and microscale particles, underscoring the risk of overgeneralizing from nanoscale-only designs [13, 16].

Larger microplastic particles may interact with the intestinal barrier through mechanisms distinct from those of nanoplastics. While they are less likely to undergo efficient cellular internalization, larger particles may influence intestinal physiology through interactions with the mucus layer, localized epithelial irritation, or indirect effects on the gut microbial environment. Product-derived and foodborne exposure studies further suggest that larger or irregular particles may remain biologically relevant even when their behavior differs from the highly internalizable spherical nanoparticles often used in mechanistic systems [19, 21, 23].

In addition, most experimental studies employ relatively uniform particles, whereas environmental plastics typically exist as irregular fragments or fibers with heterogeneous surface topographies. Particle geometry, together with particle size, influences diffusion behavior within the intestinal mucus layer as well as the formation and orientation of the protein corona at the particle–biological interface. These characteristics may influence cellular access and downstream responses independently of particle size, further indicating that size cannot be interpreted in isolation when comparing biological effects across experimental systems [20, 23, 27].

Taken together, the evidence indicates that the relationship between particle size and biological response is outcome- and model-dependent rather than uniformly size-driven. Nanoscale particles provide important mechanistic information on cellular internalization, intracellular localization, and downstream stress responses, but the limited representation of larger and environmentally heterogeneous microplastics creates a “size gap” between mechanistic research and realistic exposure conditions. Future studies should therefore incorporate broader particle size distributions and irregular environmental fragments while evaluating particle size together with polymer composition, morphology, surface characteristics, exposure conditions, and model architecture.

### From pristine particles to complex biological interfaces

Surface chemistry and the resulting interfacial charge represent critical determinants of micro- and nanoplastic (MNPL) behavior within the gastrointestinal microenvironment. The evidence map, summarized by particle-condition categories in Fig. 5, shows a substantial reliance on pristine particles (68.9%, *n* = 62/90). While the use of pristine particles facilitates the investigation of baseline toxicological responses under controlled laboratory conditions, it also provides a simplified representation of the complex physicochemical environment of the mammalian gut.

By contrast, studies using environmentally or physiologically transformed particles remained limited. Digestion-modified, aged or weathered, and product-released particles together represented less than one-third of the included evidence base. Taken together, these distributions indicate that the current experimental literature remains strongly weighted toward simplified particle preparations, despite the increasing recognition that transformation history can substantially alter biological behavior. This issue is underscored by studies using product-released particles from food containers and infant feeding bottles, as well as digestion-modified foodborne PET particles, all of which showed that transformed materials can induce intestinal stress responses that differ from those observed with conventional pristine particles [19, 21, 23, 24]. Recent mechanistic studies further link intestinal MNPL exposure to ROS-dependent inflammatory signaling, mitochondrial–AMPK–DNA damage pathways, and mitochondrial bioenergetic dysfunction, reinforcing the close coupling between oxidative stress, mitochondrial injury, inflammation, and genotoxic responses [21, 23, 24]. These findings indicate that particle transformation should be considered not merely as a secondary physicochemical characteristic but as an important source of variation when interpreting intestinal toxicological responses. However, the relatively small number of studies using aged, weathered, digestion-modified, or product-released particles limits direct comparison with pristine materials and prevents a general conclusion that transformed particles are consistently more or less toxic. Instead, the available evidence supports the need to evaluate transformation history as a determinant of particle behavior and biological response alongside polymer type, particle size, and exposure conditions.

In physiological media, particle surface chemistry does not operate in isolation. Instead, it acts as a key determinant of biomolecular adsorption and protein corona formation, which can redefine a particle’s biological identity before it reaches the intestinal epithelium. Surface characteristics may also influence interactions with the intestinal mucus barrier. For example, electrostatic properties can alter retention within the negatively charged mucin network, thereby modulating particle transport, epithelial access, and local residence time. Consequently, simplified experimental systems using pristine particles may not fully capture the electrostatic and biomolecular interactions that occur in vivo.

The underrepresentation of environmentally transformed particles is particularly important because MNPLs are rarely pristine outside laboratory settings. In natural and consumer-related environments, they undergo ultraviolet exposure, oxidation, abrasion, digestion-related modification, and release from polymer-based products. These processes alter surface roughness, functional group composition, adsorption capacity, and biological reactivity. Yet such realistic surface states remain comparatively uncommon in experimental intestinal toxicology. Recent digestion-modified and transformation-focused studies further suggest that these altered particle states can influence mitochondrial stress, barrier dysfunction, oxidative injury, and bioenergetic collapse, emphasizing that transformation history is not simply a contextual variable but a mechanistically important determinant of intestinal toxicity [23, 24].

Taken together, the evidence indicates that particle condition and interfacial properties should be considered integral components of intestinal MNPL toxicity rather than secondary experimental descriptors. Future studies should increasingly incorporate aged, weathered, digestion-modified, and product-released particles, together with systematic characterization of their surface properties, to improve the ecological and physiological relevance of experimental models and to better reflect the particle states encountered at the human gastrointestinal interface.

### Gut microbiota interactions: an emerging but underexplored axis

The evidence map identified gut microbiota and probiotic interactions as a distinct but comparatively underrepresented dimension of intestinal MNPL research. Nine studies addressed microbiota composition, microbial diversity or function, probiotic viability, or probiotic-mediated modulation of particle-associated intestinal responses. These studies encompassed human-derived fecal or simulated microbiota systems, intestinal epithelial–microbiota models, and probiotic-associated experimental configurations, indicating that microbiota-related responses are increasingly being incorporated into intestinal MNPL research but remain substantially less investigated than epithelial barrier dysfunction, oxidative stress, and cellular injury.

Importantly, the available studies suggest that microbiota-related effects are not limited to changes in microbial abundance or diversity. The reported evidence encompasses alterations in microbial function, probiotic viability, particle-associated microbial interactions, and modulation of intestinal responses in combined epithelial–microbial systems. For example, human-derived fecal and simulated gut microbiota models have identified changes in microbial composition or function following exposure to PE, PET, and product-derived micro- and nanoplastics, while probiotic-associated studies have examined whether selected bacterial strains can influence particle-associated responses or interact directly with plastic particles. The available evidence therefore supports viewing the gut microbiota as a potential biological interface through which MNPL exposure may influence intestinal physiology rather than as an isolated secondary endpoint.

A key limitation, however, is the heterogeneity of the microbiota models and exposure configurations. The available studies differ substantially in the use of human versus animal-derived microbiota, isolated bacterial cultures versus complex microbial communities, polymer type, particle size, exposure duration, and co-exposure conditions. This heterogeneity currently prevents a consistent conclusion regarding whether particular polymers or particle sizes exert reproducible microbiota-specific effects. Instead, the evidence points to a context-dependent interaction in which particle characteristics, microbial community composition, and the surrounding intestinal environment may jointly determine the biological response.

Future investigations should therefore integrate microbiota-inclusive intestinal models with epithelial, immune, and barrier endpoints rather than evaluating microbial changes in isolation. Human-derived microbiome systems, intestinal epithelial–microbiota co-cultures, organoid-based platforms, and dynamic gut-on-a-chip models may provide complementary approaches for determining whether microbiota alterations contribute directly to barrier dysfunction, inflammatory signaling, metabolic disturbance, or altered particle bioavailability. Standardization of microbiota composition, exposure conditions, particle characterization, and microbial outcome measures will also be essential for improving cross-study comparability and establishing the translational relevance of microbiota-mediated MNPL effects.

### Human health relevance and environmental exposure context

The translational relevance of the reported experimental findings requires careful consideration of the exposure conditions used across the included studies. As summarized in Supplementary Table S1, exposure concentrations varied substantially among experimental systems, and many studies reported concentrations as “Variable” rather than providing a directly comparable numerical dose. Where numerical concentrations were reported, examples included 1–100 µg/mL, 10–100 µg/mL, 1–1000 µg/mL, and, in some experimental configurations, substantially higher concentrations such as 68 mg/mL. In vivo studies used different exposure metrics, including 0.1–1 g/day in porcine models. These differences illustrate the substantial heterogeneity of exposure conditions across the evidence base and indicate that the biological responses observed in individual studies should not be interpreted as representing a uniform human exposure level.

Importantly, these experimental concentrations cannot be directly equated with real-world human exposure estimates because in vitro studies generally express exposure as a concentration in the culture medium, whereas human exposure is determined by the amount of particles encountered or ingested over time and is influenced by absorption, gastrointestinal transit, particle retention, and elimination. Available human exposure assessments illustrate the large difference in exposure metrics and the uncertainty surrounding current estimates. For example, the World Health Organization estimated that microplastic intake from drinking water based on available occurrence data could be approximately 0.01–2 µg/day, whereas an intentionally conservative scenario yielded an estimated intake of 85 µg/day; the latter was considered highly unlikely because it combined extreme assumptions regarding particle characteristics and exposure [29]. Similarly, a recent systematic review of dietary exposure estimates reported a very broad range of 0.0002–1,531,524 microplastic particles/day across food and drinking-water studies, with substantial variation attributable to food type, analytical methods, particle characteristics, and exposure assumptions [28]. These estimates highlight that current human exposure assessments remain highly uncertain and are generally expressed using metrics that are not directly comparable with the µg/mL concentrations commonly used in vitro. Therefore, the experimental concentrations represented in the present evidence map should primarily be interpreted as concentrations used to investigate biological responses under controlled conditions rather than as direct estimates of human exposure.

The environmental relevance of exposure is further influenced by particle characteristics and transformation state. As discussed above, the evidence base was dominated by virgin particles, whereas aged or weathered, digestion-modified, and product-released particles were considerably less represented. This pattern is important for human health interpretation because environmentally encountered particles may differ from commercially manufactured reference particles in size distribution, shape, surface chemistry, aggregation state, biofilm formation, and interactions with food components or co-contaminants. Consequently, biological responses observed with pristine and highly characterized particles may provide valuable mechanistic information but cannot automatically be assumed to reflect responses to environmentally transformed particles reaching the human gastrointestinal tract.

Taken together, the evidence does not currently support a simple quantitative translation from experimental concentrations to human health risk. Instead, the main translational gap identified by this review lies in the limited integration of realistic exposure conditions with physiologically representative intestinal models. Future studies should therefore prioritize exposure scenarios that reflect environmentally encountered particle characteristics, realistic concentrations and durations, and relevant food or gastrointestinal matrices, while incorporating mucus, microbiota, immune components, and dynamic intestinal conditions where feasible. Such approaches would help determine which biological effects observed under controlled experimental conditions remain relevant under more realistic human exposure scenarios and would provide a stronger basis for future hazard and risk assessment.

## Limitations

Several limitations should be considered when interpreting the findings of this scoping review. *First*, although a systematic search strategy was applied across major scientific databases, the synthesis was limited to peer-reviewed studies indexed within the selected repositories. Consequently, potentially relevant evidence from gray literature sources—such as governmental reports, technical documents, or preprint studies—may not have been captured. As a result, some emerging toxicological evidence regarding environmentally relevant polymers may remain outside the scope of the present mapping. *Second*, the current evidence base is characterized by substantial methodological heterogeneity. The 90 included studies vary widely in terms of particle morphology, exposure concentrations, experimental models, exposure durations, and biological endpoints. While this diversity reflects the rapidly evolving nature of MNPL toxicology, it limits direct cross-study comparability and precludes the possibility of performing a formal quantitative synthesis or meta-analysis. The lack of standardized exposure protocols remains a significant obstacle for establishing consistent toxicological benchmarks, including no-observed-adverse-effect levels (NOAELs) for intestinal plastic exposure. *Third*, a notable reporting gap was identified in the physicochemical characterization of particles used in experimental systems. In many studies, particle size information was reported only in broad categorical terms rather than as precise numerical size values or distributions. This inconsistency in reporting necessitated the use of occurrence-based counting approaches to map the distribution of particle characteristics across the literature. While this strategy enables the identification of dominant research trends, it may not fully capture the complex relationships between specific particle properties and biological outcomes. Finally, in accordance with the PRISMA-ScR framework, the objective of this study was to map the breadth and characteristics of the available evidence rather than to perform a formal risk-of-bias assessment. Nevertheless, the absence of a formal risk-of-bias assessment means that the present review cannot determine the relative methodological quality or certainty of individual study findings; the quality-related observations presented in the Discussion should therefore be interpreted as reporting and methodological patterns rather than formal quality ratings. Therefore, the findings presented here should be interpreted primarily as a structural overview of the current research landscape rather than a quantitative evaluation of the certainty of toxicological effects. Despite these limitations, the systematic synthesis of 90 studies provides a comprehensive overview of the experimental strategies currently used to investigate intestinal interactions of micro- and nanoplastics in mammalian systems. This mapping highlights critical methodological gaps and may help guide the design of more standardized, environmentally relevant, and physiologically representative studies in future intestinal MNPL research. Furthermore, evidence from simulated intestinal microbial ecosystems and probiotic-based experimental approaches highlights the relevance of microbiota-associated responses to micro- and nanoplastic exposure [35, 36].

## Conclusions

This scoping review maps the experimental landscape of intestinal micro- and nanoplastic (MNPL) research across 90 peer-reviewed studies and identifies substantial methodological concentration within the current evidence base. In vitro studies predominated (88.9%, *n* = 80/90), with Caco-2-based systems representing the most frequently used experimental platform (52.2%, *n* = 47/90) and Caco-2 monoculture-like configurations accounting for 36.7% (*n* = 33/90). Polystyrene was also strongly overrepresented, appearing in 60.0% of the included studies (*n* = 54/90). These patterns indicate that current mechanistic understanding is derived largely from simplified intestinal models and a restricted range of polymer types, limiting environmental and physiological representativeness.

Substantial heterogeneity in particle characterization and exposure reporting further limits cross-study comparability. In particular, inconsistent reporting of particle size, transformation state, and exposure conditions constrains the interpretation of biological responses across experimental systems. Future research should therefore prioritize physiologically representative intestinal models, including organoid and organ-on-a-chip platforms, environmentally aged or digestion-modified particles, and standardized physicochemical characterization and exposure protocols.

Overall, the current evidence provides a strong mechanistic foundation for intestinal MNPL toxicology but remains insufficiently standardized and environmentally representative for robust translational interpretation. The evidence map developed here identifies priority methodological gaps and provides a framework for designing more realistic, comparable, and physiologically relevant studies of intestinal MNPL effects.

## Supplementary Information

Below is the link to the electronic supplementary material.

Supplementary Material 1

## Data Availability

The datasets supporting the conclusions of this article are included within the article and its additional files.

## Declarations

### AI Declaration

ChatGPT (OpenAI) was used to generate the schematic illustration presented in Fig. 6 to visually communicate the findings synthesized from the included studies. Both authors critically reviewed and verified the scientific content, biological mechanisms, and figure labels for consistency with the reviewed literature. The authors take full responsibility for the scientific accuracy and integrity of the final illustration.
