# Supplementary material for: Mapping the biological effects of micro- and nanoplastics in mammalian intestinal models: a scoping review
Source: BMC Gastroenterol. 2026 Oct 27;26:705. doi: 10.1186/s12876-026-05475-0 (PMC13711986; doi:10.1186/s12876-026-05475-0)
Supplement: Supplementary file 1 [file 12876_2026_5475_MOESM1_ESM.docx]

**Table S1.** Detailed characteristics of the 90 studies included in the scoping review

| **Ref.** | **Study Type** | **Experimental Model** | **Polymer**  **Type** | **Particle**  **Size** | **Particle Condition** | **Exposure Scenario** | **Dose Concentration** | **Exposure Duration** | **Main**  **Endpoints** | **Main**  **Findings** |
| --- | --- | --- | --- | --- | --- | --- | --- | --- | --- | --- |
| [1] | In vitro | Caco-2 | PTFE | Micro/nano | Virgin | Single exposure | Variable | 24 - 48 h | Oxidative stress, genotoxicity, mitochondrial damage | Demonstrated that PTFE particles induce significant genotoxicity and oxidative stress. |
| [2] | In vitro | Caco-2 | PS | Nano | Virgin | Co-exposure | Variable | 24 h | Cell painting phenomics, metabolic changes | Showed that PFOS targets the cytoskeleton while nanoplastics primarily target mitochondria. |
| [3] | In vitro | Differentiated Caco-2 | PS | 60 nm, 1 µm | Digested | Digestion-simulated exposure | 68 mg/mL | 24 h | Digesta interaction, cell permeability | Explored how in vitro digestion and food matrices affect intestinal transfer of MPs. |
| [4] | In vitro | Caco-2, HT-29 | PLA | ~116 nm | Released from product | Product-derived exposure | Variable | 24 h | Internalization, barrier disruption | Quantified real-life PLA nanoplastic release from commercial teabags and observed high uptake. |
| [5] | In vitro | Intestinal cells (Crohn's susceptibility) | PET, PLA | Nano | Aged / weathered | Disease-context exposure | Variable | 24 h | Cytotoxicity, inflammation | Showed weathered nanoplastics exert enhanced toxicity in Crohn's disease models. |
| [6] | In vitro | iPSC-derived intestinal epithelia | PET, PVC, PP, PA | Variable | Virgin / Mixed | Single exposure | Variable | 24 h | Barrier disruption, IL-6/IL-8, protein corona | Correlated the pro-inflammatory protein corona of true-to-life MPs with barrier disruption. |
| [7] | In vitro | Triple culture (inverted) | PE | Micro | Virgin | Disease-context exposure | Variable | 24 h | Barrier integrity, inflammation | Validated an inverted triple-culture model for buoyant PE particles in inflamed states. |
| [8] | In vitro | Triple culture | PS, PVC | Micro/nano | Virgin | Disease-context exposure | Variable | 24 h | Cytokine release, DNA damage | Investigated acute effects of PS and PVC in mimicking healthy versus inflamed human intestine models. |
| [9] | In vitro | Digestion system + Probiotics | PE, PP, PET, PVC | Variable | Digested | Digestion-simulated exposure | Variable | Variable | Probiotic viability, particle surface | Assessed physical changes of polymers post-digestion and their direct impact on probiotics. |
| [10] | In vitro | Human intestinal organoids (with M cells) | PS | Micro/nano | Virgin | Single exposure | Variable | 24 - 48 h | Particle uptake, M cell function | Demonstrated that M cells act as sensors and specific transporters for larger plastic particles. |
| [11] | In vitro / In vivo | Colon cells, Mice | PS | 100 nm, 10 µm | Virgin | Single exposure | Variable | Variable | Ferroptosis, metabolic reprogramming | Found 100 nm NPs induce ferroptosis via endocytosis, while 10 µm MPs cause metabolic reprogramming. |
| [12] | In vitro | Tri-culture (Caco-2/HT29-MTX/Raji B) | PS | Micro/nano | Virgin | Single exposure | Variable | 24 h | Size-dependent internalization | Elucidated specific contribution and differential uptake rates of each cell type based on particle size. |
| [13] | In vitro | Caco-2 | PS | Nano | Virgin | Single exposure | Variable | 24 h | Cytotoxicity, ROS, genotoxicity | Evaluated cell penetration of PS-NPs and demonstrated ROS generation and early genotoxic effects. |
| [14] | In vitro | Small intestinal epithelium | PE, PS | 25 nm - 1 µm | Virgin | Single exposure | Variable | 24 h | Toxicity, permeability, translocation | Confirmed significant cellular uptake and internal translocation without severe acute toxicity. |
| [15] | In vitro | Tri-culture small intestinal epithelium | PE | Micro/nano | Aged / weathered | Digestion-simulated exposure | 400 µg/mL | 1 - 2 h | Fat digestion, lipid corona | Incineration-generated PE-MNPs increased fat digestion and absorption via lipid corona formation. |
| [16] | In vitro | 3D tri-culture small intestinal model | PS | 26 nm | Digested | Digestion-simulated exposure | Variable | 4 h | Translocation, TEER, LDH, ROS, mechanistic inhibition | Validated passive and active uptake mechanisms, including phagocytosis/endocytosis, in a triculture model. |
| [17] | In vitro | Caco-2 | PS | Micro | Other / mixed | Co-exposure | Variable | 24 h | Adsorption behavior, cytotoxicity | Demonstrated adsorption behavior of nonylphenol on PS-MPs and the resulting synergistic cytotoxicity. |
| [18] | In vitro | Caco-2 | PS | Micro | Other / mixed | Co-exposure | Variable | 24 h | Oxidative stress, barrier dysfunction | Pre-absorbed nonylphenol on PS-MPs generated severe oxidative stress and disrupted the intestinal barrier. |
| [19] | In vitro | Caco-2 | PS | Nano | Virgin | Co-exposure | Variable | 24 h | Metal carrier effect | Explored the Trojan horse effect by investigating how PS nanoplastics act as carriers for silver nanoparticles. |
| [20] | In vitro | Caco-2/HT29, Raji-B | PS | Nano | Virgin | Single exposure | Variable | 24 h | Viability, membrane integrity | Showed high uptake but no significant acute cytotoxic effects or barrier disruptions at 24h. |
| [21] | In vitro | Caco-2 | PS | Nano | Virgin | Single exposure | Variable | Up to 8 weeks | Long-term toxicity | Showed that prolonged sub-chronic exposure induces distinct cellular adaptations and stress. |
| [22] | In vitro | HT-29, HCT-116 | PE | Micro | Virgin | Disease-context exposure | 10 µg/mL | Up to 72 h | Metabolic reprogramming | Revealed that PE microplastics drive metabolic reprogramming in colorectal cancer cells. |
| [23] | In vitro | Human intestinal cells | PE | Nano | Virgin | Single exposure | Variable | Variable | Toxicological impact, cellular uptake | Investigated the specific toxicological impacts and internalization mechanisms of PE nanoplastics. |
| [24] | In vivo / Ex vivo | Human fecal analysis, Gut microbiota | NPs / MPs | Micro/nano | Released from product | Product-derived exposure | Variable | Variable | Exposure pathways, gut dysbiosis | Linked the extensive use of plastic take-out containers directly to specific gut microbiota dysbiosis. |
| [25] | In vitro | M-ARCOL gut model | PE | Micro | Virgin | Single exposure | Variable | Variable | Gut microbiota composition | Repeated exposure to PE MPs induced adult donor-dependent effects in human gut microbiota. |
| [26] | In vivo | Porcine ileum | PET | Micro | Virgin | Single exposure | 0.1 - 1 g/day | 28 days | Neurotransmitters, cytokines | Investigated the effect of PET microparticles on the enteric nervous system and cytokines. |
| [27] | In vivo | Porcine fecal microbiome | PET | Micro | Virgin | Single exposure | 0.1 - 1 g/day | 28 days | Microbiome diversity | Analyzed the impact of different dosages of PET microparticles on the porcine intestinal microbiome. |
| [28] | In vitro | Pathogenic and probiotic bacteria | PS | 100 nm - 5µm | Virgin | Single exposure | 0.1 - 100 mg/L | Variable | Bacterial growth, V. cholerae proliferation | Promoted V. cholerae reproduction while inhibiting the growth of probiotic strains. |
| [29] | In vitro | Gut-on-a-chip | PS | 100 nm | Virgin | Single exposure | 50–200 µg/mL | 24 h | Cytokines, tight junctions, apoptosis | Revealed that enhanced peristalsis reduces nanoplastic-induced intestinal inflammation. |
| [30] | In vitro | Caco-2, HT-29 | PE | 7–19.2 µm | Virgin / Extract | Single exposure | Variable | 48 h | Cell viability, oxidative stress | PE particles and their extracts affected cell viability and increased oxidative stress. |
| [31] | In vitro | Intestinal and placental barrier models | PS | 50 nm–5 µm | Virgin | Single exposure | 1–100 µg/mL | 24 h | Cytotoxicity, barrier integrity | Conducted a multi-endpoint toxicological assessment demonstrating no acute cytotoxicity but high cellular uptake. |
| [32] | In vitro | Human intestinal organoids | PS | 100 nm | Virgin | Single exposure | 10–100 µg/mL | 24 h | Cellular accumulation, endocytosis, apoptosis | Demonstrated distinct accumulation of nanoplastics in goblet and Paneth cells in organoid models. |
| [33] | In vitro | Caco-2, Gut microbiota | PE | 30–50 µm | Virgin | Co-exposure | Variable | 24 - 48 h | Cytotoxicity, gut microbiota changes | Co-exposure exacerbated TBBPA cytotoxicity and disturbed key gut microbial pathways. |
| [34] | In vitro | Caco-2 | PET, PS | Micro/nano | Released from product | Product-derived exposure | Variable | 24 h | Membrane damage, oxidative stress | Demonstrated that plastic takeaway food containers release massive MPs during thermal usage, causing toxicity. |
| [35] | In vitro | Tri-culture small intestinal epithelium | PS | 25 nm–1 µm | Virgin | Co-exposure | Variable | 24 h | Bioavailability, absorption | Ingested PS-MNPs significantly increased the absorption of co-ingested arsenic and boscalid. |
| [36] | In vitro | Duodenum-chip (Healthy & Crohn's) | PS | 25 nm | Virgin | Disease-context exposure | Variable | 24 h | Uptake mechanisms, toxicity | Studied active and passive uptake mechanisms in a disease-context duodenum-chip model without acute toxicity. |
| [37] | In vitro | Caco-2 + Probiotics | PS | Nano | Virgin | Co-exposure | Variable | 18 h | Particle internalization, permeability | Specific probiotic strains suppressed nanoplastic transcellular permeability and internalization. |
| [38] | In vitro | Caco-2 | PS | 50 nm | Virgin | Single exposure | 1–100 µg/mL | 24 - 48 h | DNA repair response, ROS, apoptosis | Revealed that polystyrene nanoparticles lead to dysfunction in DNA base excision repair (BER) mechanisms. |
| [39] | In vitro | Caco-2 | PE | 1–10 µm | Virgin | Co-exposure | Variable | 24 h | Oxidative damage, inflammation | Apple extract prevented oxidative and inflammatory damage caused by environmental PE microplastics. |
| [40] | In vitro | 3D Intestine barrier + Immune cells | Polyolefins, Tire wear | 50–500 µm | Virgin | Single exposure | Variable | 24 h | Immune response, toxicity | Developed a 3D barrier model with macrophages to assess the effects of tire wear and polyolefins. |
| [41] | In vitro | Simulated digestive tract + Caco-2 | NPs | Nano | Digested | Digestion-simulated exposure | Variable | Variable | Toxicity, combined effects, protein corona | Evaluated the impact of food matrices on the characteristics and toxicity of ingested nanoplastics. |
| [42] | In vitro | Caco-2 | PS | 0.1–1.0 µm | Digested | Digestion-simulated exposure | Variable | 24 h | Toxicity, transport function | Showed that the digestive process alters PS-MP toxicity and increases pro-inflammatory effects via corona formation. |
| [43] | In vitro | Caco-2 | PS | Nano/micro | Virgin | Co-exposure | Variable | 24 h | Particle uptake, cytotoxicity | PFOS enhanced the cellular uptake of PS particles, while PS particles weakened PFOS cytotoxicity. |
| [44] | In vitro | Tri-culture small intestinal epithelium | PS | Nano | Aged / weathered | Single exposure | Variable | 24 h | Toxicity, bioavailability | Investigated the impact of UV aging on the toxicity and bioavailability of ICP-MS traceable nanoplastics. |
| [45] | In vitro | Caco-2 | Micro(bio)plastics | Micro | Released from product | Product-derived exposure | Variable | 24 h | Viability, ROS generation | Compared plant-based and petroleum-derived microplastics, showing bio-plastics offer potential protection against ROS. |
| [46] | In vitro | Caco-2 mono and tri-culture | PS | 50 nm, 500 nm | Virgin | Single exposure | Variable | Variable | Cytotoxicity, translocation | Assessed particle association and translocation in increasingly complex intestinal cell models without barrier impact. |
| [47] | In vitro | Human (HRT-18) and Murine (CMT-93) | PS | Micro | Virgin | Single exposure | Variable | 6 - 48 h | Inflammation, cytotoxicity | Evaluated pro-inflammatory and cytotoxic effects across both human and murine intestinal cell lines. |
| [48] | In vitro | Caco-2, HT29MTX (2D/3D) | HDPE | Nano | Virgin | Co-exposure | Variable | 24 h | Toxicity, barrier integrity | Investigated the synergistic toxicity of high-density polyethylene NPs in combination with silver NPs. |
| [49] | In vitro | HT-29, Caco-2, HepG2 | PS | 30, 50, 100 nm | Virgin | Single exposure | Variable | 24 h | Uptake mechanism, toxicity | Studied size-dependent uptake mechanisms and toxicity emphasizing clathrin-mediated endocytosis. |
| [50] | In vivo / In vitro | Mice / Intestinal cells | NPs | Nano | Aged / weathered | Single exposure | Variable | Variable | Barrier homeostasis, remediation | Explored barrier dysfunction induced by aged NPs and mitigation through dietary quercetin intervention. |
| [51] | In vitro | Caco-2 | PE, PET | Micro | Virgin | Single exposure | Variable | Variable | Proliferation, apoptosis, autophagy | Unraveled proliferation, apoptosis, and autophagy activation following environmental MP exposure. |
| [52] | In vivo / In vitro | CRC tissue, HCT-116, mice | PS | Micro/nano | Virgin | Disease-context exposure | 10 µg/mL | 24 - 48 h | Chemoresistance, tumor progression | MPs accumulate in CRC tissues and promote oxaliplatin resistance via autophagy and mTOR. |
| [53] | In vitro | Caco-2, HepaRG | PLA, PMMA, MF | 25 nm - 2 µm | Virgin | Single exposure | 1 - 1000 µg/mL | 24 h | Cell viability, ROS, particle uptake | Demonstrated subcellular detection and oral bioavailability of food-relevant nanoplastics. |
| [54] | In vitro | 3D co-culture | PLA, PMMA, MF | 25 nm - 2 µm | Virgin | Single exposure | 1 - 1000 µg/mL | 24 h | Translocation, hepatic transfer | Highlighted M-cell and mucus-dependent uptake and downstream hepatic transfer. |
| [55] | In vitro | Caco-2 | PLA, PMMA, MF | 25 nm - 2 µm | Digested | Digestion-simulated exposure | 10 - 1000 µg/mL | 24 h | Aggregation, uptake, transport | Demonstrated that digestive protein corona formation modifies particle toxicity. |
| [56] | In vitro | Caco-2 | PS | 2 µm | Virgin | Single exposure | Low concentration | Up to 12 days | Bioenergetic effects, mitochondria | Long-term exposure showed bioenergetic effects depending on cell differentiation. |
| [57] | In vitro | Caco-2 | PS | 100 nm | Virgin | Single / Repeated | 10^2^−10^9^ particles/mL | Up to 12 days | Glycolysis, mitochondrial health | Low-dose repeated exposure exacerbated mitochondrial dysfunction and evoked glycolysis. |
| [58] | In vitro | Caco-2, Lactobacillus | PS | Nano | Virgin | Co-exposure | 0.1 mg/mL | Variable | Bioremoval, MTT assay | Selected Lactobacillus strains demonstrated high percentages of nanoplastic bioremoval. |
| [59] | In vitro | SHIME | PS | 1 µm | Virgin | Single exposure | 50 - 500 µg/mL | 24 h | Gut microbiota, SCFAs | MPs altered gut microbiota, reduced beneficial bacteria, and impacted intestinal cells. |
| [60] | In vitro | Caco-2, HepG2 | Mixed plastics | Micro | Aged / weathered | Single exposure | Variable | 24 - 48 h | Genotoxicity, DNA damage | Assessed the genotoxicity of particles from grinded plastic items without acute toxicity. |
| [61] | In vitro | Caco-2 | PS | Micro | Virgin | Single exposure | Variable | 24 h | Mitochondrial stress, redox | Polystyrene microplastics of varying sizes and shapes induced distinct stress responses. |
| [62] | In vitro | SNU-1826 | PS | 0.4 - 0.6 µm | Virgin | Single exposure | Variable | 24 h | Autophagy, ER stress | Examined human colon cells response to autophagy and ER stress during internalization. |
| [63] | In vivo / In vitro | L. plantarum, Mice | PS | Micro | Virgin | Co-exposure | 100 µg/mL | Variable | Bile acid metabolism | L. plantarum reduced MP toxicity by regulating bile acid metabolism and particle binding. |
| [64] | In vitro | IEC-6 | Mixed MPs | Micro | Aged / weathered | Single exposure | Variable | Variable | Lipid dysregulation | Multi-polymer real-world microplastics adhered to IEC-6 cells and induced dysregulation. |
| [65] | In vitro | Colonocytes | PS | 100 nm | Virgin | Co-exposure | Variable | 24 h | Cytotoxicity, apoptosis | Fluoride presence enhanced the cytotoxicity of amino-modified polystyrene nanoparticles. |
| [66] | In vitro | Caco-2, HepG2 | PE, PP, PET, PVC | Micro | Virgin | Single exposure | 100 - 1000 µg/cm2 | 24 - 48 h | Uptake, cytotoxicity | Resolved polymer bias by comparing environmentally relevant plastics (PE, PP, PET, PVC). |
| [67] | In vitro | Gut/liver models | Micro/nano | Variable | Virgin | Single exposure | Variable | 24 h | Dispersant toxicity | Revealed methodological flaws caused by dispersant-driven apparent toxicity. |
| [68] | In vitro | Caco-2, HepaRG | PS | Micro/nano | Virgin | Single exposure | Variable | 24 h | ABC transporters | Showed lack of significant effects of PS particles on activity of human drug transporters. |
| [69] | In vitro | Epithelial cells | PS | Nano | Virgin | Single exposure | Variable | 24 h | High-throughput toxicity | Conducted high-throughput toxicological assessment of nanoplastics. |
| [70] | In vitro | Caco-2 | PS | Nano | Digested | Digestion-simulated exposure | Variable | 24 h | Protein corona, cytotoxicity | Investigated the behavior of PS-NPs in real food matrices during simulated digestion. |
| [71] | In vitro | HCT116 | PS | Nano | Virgin | Single exposure | Variable | Variable | Genotoxicity, oxidative stress | Genotoxicity and oxidative stress induction by PS nanoparticles in colorectal cancer cells. |
| [72] | In vitro | HT-29 | PS | 3, 10 µm | Virgin | Single exposure | Variable | Up to 48 days | ROS, long-term stress | Demonstrated the differential impact of sub-chronic versus acute exposure to MPs. |
| [73] | In vitro | Caco-2 | PS | Micro/nano | Virgin | Co-exposure | Variable | 24 h | Oxidative stress | Assessed the protective effects of catechin against size-dependent MP cytotoxicity. |
| [74] | Organ-on-a-chip | Gut-liver-on-a-chip | PS | 100 nm | Virgin | Peristaltic flow | Variable | 24 h | Organ cross-talk, hepatic transfer | Demonstrated the critical role of intestinal peristalsis in mitigating liver damage. |
| [75] | In vitro | Caco-2 | PGA, PLA, PBS | Micro | Released from product | Single exposure | Variable | Variable | Intracellular distribution | Assessed the environmental release behavior and toxicity of novel biodegradable plastics. |
| [76] | In vitro | Caco-2 | PS | 0.1, 5 µm | Virgin | Single exposure | Variable | 24 h | Efflux pump inhibition | Analyzed size-dependent effects of PS-MPs on cytotoxicity and efflux pump inhibition. |
| [77] | In vitro | Caco-2 | PS | Micro | Virgin | Single exposure | Variable | Variable | Transcriptomic profile | Identified responsible genes and involved pathways contributing to PS-MPs toxicity. |
| [78] | In vitro | Caco-2 | PP, PS | Micro/nano | Released from product | Product-derived exposure | Variable | Variable | Thermal release, cell stress | Established thermal release of MPs from real-world food containers and linked to stress. |
| [79] | Multi-model | RKO, HT-29, HCT-116, HIEC-6, Mice | PS | 100 nm | Virgin | Multi-model exposure | Variable | 24 - 48 h | Growth/survival, intracellular accumulation, autophagic flux | Identified impaired autophagic flux as a major toxicity mechanism across normal and tumor-derived intestinal cells. |
| [80] | In vitro | Caco-2 human intestinal cells | PP | Irregular | Released from product | Product-derived exposure | Working solution (20%) | 12 h | ROS, lipid peroxidation, IL-6, TNF-α, inflammasome signaling | Linked infant feeding bottle-derived MPs to intestinal inflammation through the ROS/NLRP3/Caspase-1/IL-1β axis. |
| [81] | In vitro | Caco-2 | PS | Micro/nano | Virgin | Co-exposure | Variable | 24 h | Cytotoxicity, apoptosis | Polystyrene nanoplastics promoted okadaic acid-induced apoptosis more significantly than microplastics. |
| [82] | In vivo | Mice (DSS-induced UC model) | PET | 5 µm | Virgin | Disease-context exposure | 0.5 mg/mouse/day | 7 days | Multi-omics, hub genes | Demonstrated that PET-MPs aggravate pre-existing colitis and provided disease-context mechanistic evidence. |
| [83] | In vitro | Caco-2 | NPs | Nano | Virgin | Co-exposure | Variable | 24 h | Toxicity, autophagy mitigation | Nobiletin-mediated autophagy mitigated nanoplastic-induced toxicity in human intestinal cells. |
| [84] | In vitro | Caco-2 | PS | Micro | Aged / weathered | Single exposure | Variable | Variable | Cytotoxicity, functional groups | Photo-transformation changed the surface morphology of PS-MPs and enhanced their cytotoxicity. |
| [85] | In vitro | Human intestinal epithelial co-culture | PET | Diet-relevant PET-MPs | Digested | Digestion-simulated exposure | 50 - 200 µg/mL | 24 h | Cytotoxicity, oxidative stress, barrier dysfunction, AMPK, DNA damage | Discovered the mitochondrial-AMPK-DNA damage pathway as a central mechanism of PET-MP intestinal injury. |
| [86] | In vitro | Human intestinal co-culture / composite food-digesta model | PET | Predominantly 20–100 µm fraction | Digested | Digestion-simulated exposure | Variable | Variable | Mitochondrial respiration, oxidative stress, metabolic profiling | Detailed the hyperactivation of mitochondrial Complex I as a driver of intestinal bioenergetic collapse. |
| [87] | In vitro | Caco-2 | PS | Nano | Aged / weathered | Co-exposure | 400 µg/mL | 24 h | Cytotoxicity, properties changes | Aging amplified the combined toxic effects of PS-NPs and norfloxacin on human intestinal cells. |
| [88] | In vitro | Caco-2 monolayer | PS | 50 nm | Virgin | Single exposure | 10^12^−10^14^ part/mL | 6 - 18 h | ROS, membrane integrity, SERS | Characterized nanoplastics-induced stress and its SERS fingerprint in an intestinal membrane model. |
| [89] | In vitro | CCD841CoN, HIEC-6 | PS | 0.1 - 5 µm | Virgin | Single exposure | 12.5 - 100 µg/mL | 24 - 48 h | Cytotoxicity, mitochondrial | Revealed specific uptake and toxicity differences between normal and cancer intestinal cell lines. |
| [90] | In vitro | Caco-2 intestinal barrier / Transwell model | PET | Micro | Virgin | Co-exposure | PFOA 100 µg/mL + PET MPs 50 or 250 µg/mL | 24 h | TEER, tight junctions, ROS, MMP, LDH | Showed synergistic barrier disruption and tight-junction injury during PET-MP/PFOA co-exposure. |

**References**

1.	Abass, D., et al., *Polytetrafluoroethylene (PTFE, Teflon) microplastics and nanoplastics induce oxidative stress, mitochondrial damage, and genotoxicity in human intestinal cells.* Journal of Hazardous Materials, 2025.

2.	Alijagic, A., et al., *Nanoplastics drive toxicity under co-exposure with perfluorooctanesulfonic acid in human intestinal cells.* Environmental Chemistry Letters, 2025.

3.	Arranz, E., et al., *Following the fate of polystyrene micro and nanobeads during in vitro digestion.* Current Research in Food Science, 2025.

4.	Banaei, G., et al., *The release of polylactic acid nanoplastics (PLA-NPLs) from commercial teabags. Obtention, characterization, and hazard effects of true-to-life PLA-NPLs.* Journal of Hazardous Materials, 2023.

5.	Bard, V., et al., *Toxicity of polyethylene terephthalate and polylactic acid nanoplastics, pristine and weathered in environmentally-relevant conditions, to human intestinal cells representative of genetic susceptibility to Crohn's disease.* Food and Chemical Toxicology, 2026.

6.	Brouwer, H., et al., *Toxicity of true-to-life microplastics to human iPSC-derived intestinal epithelia correlates to their protein corona composition.* Journal of Hazardous Materials, 2025.

7.	Busch, M., et al., *Investigations of acute effects of polystyrene and polyvinyl chloride micro- and nanoplastics in an advanced in vitro triple culture model of the healthy and inflamed intestine.* Environmental Research, 2021.

8.	Busch, M., A.A.M. Kämpfer, and R.P.F. Schins, *An inverted in vitro triple culture model of the healthy and inflamed intestine: Adverse effects of polyethylene particles.* Chemosphere, 2021.

9.	Chen, H., et al., *Investigation of Microplastics in Digestion System: Effect on Surface Microstructures and Probiotics.* Bulletin of Environmental Contamination and Toxicology, 2022.

10.	Chen, Y., et al., *Biological effects of polystyrene micro- and nano-plastics on human intestinal organoid-derived epithelial tissue models without and with M cells.* Nanomedicine: Nanotechnology, Biology and Medicine, 2023.

11.	Cheng, Y., et al., *Molecular mechanism differences between nanoplastics and microplastics in colon toxicity: nanoplastics induce ferroptosis-mediated immunogenic cell death, while microplastics cause cell metabolic reprogramming.* Journal of Nanobiotechnology, 2025.

12.	Choi, H., et al., *Size-Dependent Internalization of Microplastics and Nanoplastics Using In Vitro Model of the Human Intestine—Contribution of Each Cell in the Tri-Culture Models.* Nanomaterials, 2024.

13.	Cortés, C., et al., *Nanoplastics as a potential environmental health factor: effects of polystyrene nanoparticles on human intestinal epithelial Caco-2 cells.* Environmental Science: Nano, 2020.

14.	DeLoid, G.M., et al., *Toxicity, uptake, and nuclear translocation of ingested micro-nanoplastics in an in vitro model of the small intestinal epithelium.* Food and Chemical Toxicology, 2021.

15.	DeLoid, G.M., et al., *Incineration-Generated Polyethylene Micro-Nanoplastics Increase Triglyceride Lipolysis and Absorption in an In Vitro Small Intestinal Epithelium Model.* Environmental Science & Technology, 2022.

16.	DeLoid, G.M., et al., *Mechanisms of ingested polystyrene micro-nanoplastics (MNPs) uptake and translocation in an in vitro tri-culture small intestinal epithelium.* Journal of Hazardous Materials, 2024.

17.	Ding, F., et al., *Adsorption Behavior of Nonylphenol on Polystyrene Microplastics and Their Cytotoxicity in Human Caco-2 Cells.* Water, 2022.

18.	Ding, F., et al., *Polystyrene microplastics with absorbed nonylphenol induce intestinal dysfunction in human Caco-2 cells.* Environmental Toxicology and Pharmacology, 2024.

19.	Domenech, J., et al., *Polystyrene Nanoplastics as Carriers of Metals. Interactions of Polystyrene Nanoparticles with Silver Nanoparticles and Silver Nitrate, and Their Effects on Human Intestinal Caco-2 Cells.* Biomolecules, 2021.

20.	Domenech, J., et al., *Interactions of polystyrene nanoplastics with in vitro models of the human intestinal barrier.* Archives of Toxicology, 2020.

21.	Domenech, J., et al., *Long-Term Effects of Polystyrene Nanoplastics in Human Intestinal Caco-2 Cells.* Biomolecules, 2021.

22.	Donisi, I., et al., *In vitro evidence and integrative bioinformatics identify the SGLT2-PPARγ axis as a target against polyethylene microplastic-driven metabolic reprogramming in colorectal cancer cells.* Journal of Translational Medicine, 2026.

23.	El Basset, W., et al., *Impact of polyethylene nanoplastics on human intestinal cells.* Nanotoxicology, 2024.

24.	Feng, C., et al., *Take-out containers as nano- and microplastics reservoirs: Diet-driven gut dysbiosis in university students.* Environmental Pollution, 2025.

25.	Fournier, E., et al., *Microplastics: What happens in the human digestive tract? First evidences in adults using in vitro gut models.* Journal of Hazardous Materials, 2023.

26.	Gałęcka, I. and J. Całka, *Microplastic and the Enteric Nervous System: Effect of PET Microparticles on Selected Neurotransmitters and Cytokines in the Porcine Ileum.* International Journal of Molecular Sciences, 2024.

27.	Gałęcka, I., A. Rychlik, and J. Całka, *Influence of selected dosages of plastic microparticles on the porcine fecal microbiome.* Scientific Reports, 2025.

28.	Gu, T., et al., *Distinct impact of polystyrene microplastics on six species of common pathogenic and probiotic bacteria and their boosting support to Vibrio cholerae proliferation.* Environmental Science: Processes & Impacts, 2025.

29.	Han, J., et al., *Gut‐on‐a‐Chip Reveals Enhanced Peristalsis Reduces Nanoplastic‐Induced Inflammation.* Small, 2024.

30.	Herrala, M., et al., *Micro-sized polyethylene particles affect cell viability and oxidative stress responses in human colorectal adenocarcinoma Caco-2 and HT-29 cells.* Science of The Total Environment, 2023.

31.	Hesler, M., et al., *Multi-endpoint toxicological assessment of polystyrene nano- and microparticles in different biological models in vitro.* Toxicology in Vitro, 2019.

32.	Hou, Z., et al., *Distinct accumulation of nanoplastics in human intestinal organoids.* Science of The Total Environment, 2022.

33.	Huang, W., et al., *Influence of the co-exposure of microplastics and tetrabromobisphenol A on human gut: Simulation in vitro with human cell Caco-2 and gut microbiota.* Science of The Total Environment, 2021.

34.	Jin, T., et al., *Plastic takeaway food containers may cause human intestinal damage in routine life usage: Microplastics formation and cytotoxic effect.* Journal of Hazardous Materials, 2024.

35.	Kharaghani, D., et al., *Ingested Polystyrene Micro-Nanoplastics Increase the Absorption of Co-Ingested Arsenic and Boscalid in an In Vitro Triculture Small Intestinal Epithelium Model.* Microplastics, 2025.

36.	Kharaghani, D., et al., *Toxicity and absorption of polystyrene micro-nanoplastics in healthy and Crohn’s disease human duodenum-chip models.* Journal of Hazardous Materials, 2025.

37.	Kobayashi, K., et al., *Lactobacillus delbrueckii subsp. bulgaricus 2038 and Streptococcus thermophilus 1131 suppress polystyrene nanoplastic transcellular permeability and internalization by intestinal epithelial cells.* Scientific Reports, 2026.

38.	Kustra, A., et al., *Exposure to polystyrene nanoparticles leads to dysfunction in DNA repair mechanisms in Caco-2 cells.* Biological Research, 2025.

39.	Lama, S., et al., *Oxidative and Inflammatory Damage by Environmental Polyethylene Microplastics in Caco-2 Cells Is Prevented by Polyphenol-Rich Limoncella Apple Extract.* Oxid Med Cell Longev, 2026. **2026**: p. 3136395.

40.	Lehner, R., et al., *A novel 3D intestine barrier model to study the immune response upon exposure to microplastics.* Archives of Toxicology, 2020.

41.	Li, Z., et al., *Impact of food matrices on the characteristics and cellular toxicities of ingested nanoplastics in a simulated digestive tract.* Food and Chemical Toxicology, 2023.

42.	Liu, S., et al., *Influence of the digestive process on intestinal toxicity of polystyrene microplastics as determined by in vitro Caco-2 models.* Chemosphere, 2020.

43.	Liu, Y., et al., *Perfluorooctane sulfonate (PFOS) enhanced polystyrene particles uptake by human colon adenocarcinoma Caco-2 cells.* Science of The Total Environment, 2022.

44.	Majumder, S., et al., *Impact of UV Aging on the Toxicity and Bioavailability of Inductively Coupled Plasma Mass Spectrometry (ICP-MS)-Traceable Core–Shell Polystyrene Nanoplastics in an In Vitro Triculture Small Intestinal Epithelium Model.* Toxics, 2025.

45.	Marazuela, M.D., et al., *Food packaging characterization, composition profiles and in vitro testing of micro(bio)plastics from selected petroleum- and plant-based food containers.* Journal of Hazardous Materials, 2026.

46.	Marcellus, K.A., et al., *Exposure of Polystyrene Nano- and Microplastics in Increasingly Complex In Vitro Intestinal Cell Models.* Nanomaterials, 2025.

47.	Mattioda, V., et al., *Pro-Inflammatory and Cytotoxic Effects of Polystyrene Microplastics on Human and Murine Intestinal Cell Lines.* Biomolecules, 2023.

48.	Męczyńska-Wielgosz, S., et al., *Toxicity of High-Density Polyethylene Nanoparticles in Combination with Silver Nanoparticles to Caco-2 and HT29MTX Cells Growing in 2D or 3D Culture.* Molecules, 2025.

49.	Męczyńska-Wielgosz, S., et al., *Uptake and Toxicity of Polystyrene NPs in Three Human Cell Lines.* International Journal of Molecular Sciences, 2025.

50.	Meng, X., et al., *Nanoplastics induced health risk: Insights into intestinal barrier homeostasis and potential remediation strategy by dietary intervention.* Journal of Hazardous Materials, 2024.

51.	Najahi, H., et al., *Impact of Environmental Microplastic Exposure on Caco-2 Cells: Unraveling Proliferation, Apoptosis, and Autophagy Activation.* International Journal of Environmental Research and Public Health, 2025.

52.	Pan, W., et al., *Effects of microplastics on chemo-resistance and tumorigenesis of colorectal cancer.* Apoptosis, 2025.

53.	Paul, M.B., et al., *Beyond microplastics - investigation on health impacts of submicron and nanoplastic particles after oral uptake in vitro.* Microplastics and Nanoplastics, 2022.

54.	Paul, M.B., et al., *Complex intestinal and hepatic in vitro barrier models reveal information on uptake and impact of micro-, submicro- and nanoplastics.* Environment International, 2023.

55.	Paul, M.B., et al., *Influence of artificial digestion on characteristics and intestinal cellular effects of micro-, submicro- and nanoplastics.* Food and Chemical Toxicology, 2024.

56.	Peng, M., et al., *Cellular and bioenergetic effects of polystyrene microplastic in function of cell type, differentiation status and post-exposure time.* Environmental Pollution, 2023.

57.	Peng, M., et al., *Probing Long-Term Impacts: Low-Dose Polystyrene Nanoplastics Exacerbate Mitochondrial Health and Evoke Secondary Glycolysis via Repeated and Single Dosing.* Environmental Science & Technology, 2024.

58.	Rahimi, N.R., et al., *Determination of the ability of native potential probiotic lactobacillus strains in nanoplastic bioremoval in an in-vitro Model.* Ecotoxicology and Environmental Safety, 2025.

59.	Ren, X., et al., *Microplastic Toxicity on Gut Microbiota and Intestinal Cells: Evidence from the Simulator of the Human Intestinal Microbial Ecosystem (SHIME).* Toxics, 2025.

60.	Roursgaard, M., et al., *Genotoxicity of Particles From Grinded Plastic Items in Caco-2 and HepG2 Cells.* Frontiers in Public Health, 2022.

61.	Saenen, N.D., et al., *Polystyrene Microplastics of Varying Sizes and Shapes Induce Distinct Redox and Mitochondrial Stress Responses in a Caco-2 Monolayer.* Antioxidants, 2023.

62.	Seol, A., et al., *Differential response of SNU-1826 colon cells on the autophagy, ER stress, and inflammation during the regulation of microplastic internalization.* The Journal of Toxicological Sciences, 2025.

63.	Shi, L., et al., *Lactobacillus plantarum reduces polystyrene microplastic induced toxicity via multiple pathways: A potentially effective and safe dietary strategy to counteract microplastic harm.* Journal of Hazardous Materials, 2025.

64.	Sofield, C.E., et al., *Label-free non-destructive spectroscopic detection of mixed microplastic uptake and differential effects on intestinal epithelial cells.* Journal of Hazardous Materials, 2026.

65.	Steckiewicz, K.P., et al., *Fluoride enhances polystyrene nanoparticles cytotoxicity in colonocytes in vitro model.* Chemico-Biological Interactions, 2022.

66.	Stock, V., et al., *Uptake and cellular effects of PE, PP, PET and PVC microplastic particles.* Toxicology in Vitro, 2021.

67.	Stock, V., et al., *Microplastics and nanoplastics: Size, surface and dispersant – What causes the effect?* Toxicology in Vitro, 2022.

68.	Tastet, V., et al., *Lack of effects of polystyrene micro- and nanoplastics on activity and expression of human drug transporters.* Environmental Toxicology and Pharmacology, 2024.

69.	Tolardo, V., et al., *In Vitro High-Throughput Toxicological Assessment of Nanoplastics.* Nanomaterials, 2022.

70.	Urgun, K., et al., *Exploring the role of real food matrices on the behavior and toxicity of polystyrene nanoplastics during digestion simulation.* NanoImpact, 2025.

71.	Vecchiotti, G., et al., *Genotoxicity and oxidative stress induction by polystyrene nanoparticles in the colorectal cancer cell line HCT116.* PLOS ONE, 2021.

72.	Visalli, G., et al., *Acute and Sub-Chronic Effects of Microplastics (3 and 10 µm) on the Human Intestinal Cells HT-29.* International Journal of Environmental Research and Public Health, 2021.

73.	Wang, X., et al., *Assessment of the cytotoxicity micro- and nano-plastic on human intestinal Caco-2 cells and the protective effects of catechin.* Environmental Science: Processes & Impacts, 2024.

74.	Wang, Y., et al., *Revealing transport, uptake and damage of polystyrene microplastics using a gut-liver-on-a-chip.* Lab on a Chip, 2025.

75.	Wen, L., et al., *Environmental release behavior, cell toxicity and intracellular distribution of novel biodegradable plastic materials.* Environmental Pollution, 2025.

76.	Wu, B., et al., *Size-dependent effects of polystyrene microplastics on cytotoxicity and efflux pump inhibition in human Caco-2 cells.* Chemosphere, 2019.

77.	Wu, S., et al., *Effects of polystyrene microbeads on cytotoxicity and transcriptomic profiles in human Caco‐2 cells.* Environmental Toxicology, 2019.

78.	Xia, Y. and W.-X. Wang, *Subcellular toxicity assessments of microplastics released from food containers.* Journal of Hazardous Materials, 2025.

79.	Xu, X., et al., *Autophagic response of intestinal epithelial cells exposed to polystyrene nanoplastics.* Environmental Toxicology, 2023.

80.	Xu, Z., et al., *Exposure to irregular microplastic shed from baby bottles activates the ROS/NLRP3/Caspase-1 signaling pathway, causing intestinal inflammation.* Environment International, 2023.

81.	Yan, L., et al., *Polystyrene nanoplastics promote the apoptosis in Caco-2 cells induced by okadaic acid more than microplastics.* Ecotoxicology and Environmental Safety, 2023.

82.	Yang, X., et al., *Polyethylene terephthalate microplastics exposure enhances the risk of ulcerative colitis: insights from multiomics integration, machine learning, and molecular docking reveal intestinal toxicity mechanisms.* International Journal of Surgery, 2026.

83.	Yu, J., et al., *Nobiletin‐mediated autophagy mitigates nanoplastic‐induced toxicity in human intestinal Caco‐2 cells.* The FASEB Journal, 2025.

84.	Yu, X., et al., *Photo-transformation of microplastics and its toxicity to Caco-2 cells.* Science of The Total Environment, 2022.

85.	Zhang, C., et al., *Foodborne PET Microplastic Contamination Compromises Intestinal Barrier through a Mitochondrial-AMPK-DNA Damage Pathway.* Journal of Agricultural and Food Chemistry, 2026.

86.	Zhang, C., et al., *Mitochondrial Complex I hyperactivation drives PET microplastic-induced intestinal bioenergetic collapse.* Journal of Hazardous Materials, 2026.

87.	Zhang, L., et al., *Aging amplifies the combined toxic effects of polystyrene nanoplastics and norfloxacin on human intestinal cells.* Environmental Science: Nano, 2025.

88.	Zhang, Q. and B.M. Reinhard, *Characterizing nanoplastics‐induced stress and its SERS fingerprint in an intestinal membrane model.* Nano Select, 2021.

89.	Zhang, Y., et al., *The potential effects of microplastic pollution on human digestive tract cells.* Chemosphere, 2022.

90.	Zou, X., et al., *Effects of co-exposure to microplastics and perfluorooctanoic acid on the Caco-2 cells.* Toxicology, 2025.
